# Neem leaf glycoprotein binding to Dectin-1 receptors on dendritic cell induces type-1 immunity through CARD9 mediated intracellular signal to NFκB

**DOI:** 10.1186/s12964-024-01576-z

**Published:** 2024-04-23

**Authors:** Nilanjan Ganguly, Tapasi Das, Avishek Bhuniya, Ipsita Guha, Mohona Chakravarti, Sukanya Dhar, Anirban Sarkar, Saurav Bera, Jesmita Dhar, Shayani Dasgupta, Akata Saha, Tithi Ghosh, Juhina Das, Ugir Hossain Sk, Saptak Banerjee, Subrata Laskar, Anamika Bose, Rathindranath Baral

**Affiliations:** 1 Department of Immunoregulation and Immunodiagnostics, Chittaranjan National Cancer Institute, 37, S. P. Mukherjee Road, Kolkata, West Bengal 700026 India; 2 Jubilant Biosys Limited, 96, Digital Park Rd, Yesvantpur Industrial Suburb, Bengaluru, Karnataka 560022 India; 3 Department of Clinical and Translational Research, Chittaranjan National Cancer Institute, 37, S. P. Mukherjee Road, Kolkata, West Bengal 700026 India; 4 Department of Chemistry, University of Burdwan, Burdwan, West Bengal 713104 India; 5 Department of Pharmaceutical Technology-Biotechnology, National Institute of Pharmaceutical Education and Research (NIPER),-S.A.S. Nagar, Mohali, Punjab 160062 India

**Keywords:** Dendritic cell, Dectin-1 receptor, Neem leaf glycoprotein, NFκB, IL-10, IL-12, Type 1 immune environment

## Abstract

**Background:**

A water-soluble ingredient of mature leaves of the tropical mahogany ‘Neem’ (*Azadirachta indica*), was identified as glycoprotein, thus being named as ‘Neem Leaf Glycoprotein’ (NLGP). This non-toxic leaf-component regressed cancerous murine tumors (melanoma, carcinoma, sarcoma) recurrently in different experimental circumstances by boosting prime antitumor immune attributes. Such antitumor immunomodulation, aid cytotoxic T cell (T_c_)-based annihilation of tumor cells. This study focused on identifying and characterizing the signaling gateway that initiate this systemic immunomodulation. In search of this gateway, antigen-presenting cells (APCs) were explored, which activate and induce the cytotoxic thrust in T_c_ cells.

**Methods:**

Six glycoprotein-binding C-type lectins found on APCs, namely, MBR, Dectin-1, Dectin-2, DC-SIGN, DEC205 and DNGR-1 were screened on bone marrow-derived dendritic cells from C57BL/6 J mice. Fluorescence microscopy, RT-PCR, flow cytometry and ELISA revealed Dectin-1 as the NLGP-binding receptor, followed by verifications through RNAi. Following detection of β-Glucans in NLGP, their interactions with Dectin-1 were explored in silico. Roles of second messengers and transcription factors in the downstream signal were studied by co-immunoprecipitation, western blotting, and chromatin-immunoprecipitation. Intracellularization of FITC-coupled NLGP was observed by processing confocal micrographs of DCs.

**Results:**

Considering extents of hindrance in NLGP-driven transcription rates of the cytokines IL-10 and IL-12p35 by receptor-neutralization, Dectin-1 receptors on dendritic cells were found to bind NLGP through the ligand’s peripheral β-Glucan chains. The resulting signal phosphorylates PKCδ, forming a trimolecular complex of CARD9, Bcl10 and MALT1, which in turn activates the canonical NFκB-pathway of transcription-regulation. Consequently, the NFκB-heterodimer p65:p50 enhances *Il12a* transcription and the p50:p50 homodimer represses *Il10* transcription, bringing about a cytokine-based systemic-bias towards type-1 immune environment. Further, NLGP gets engulfed within dendritic cells, possibly through endocytic activities of Dectin-1.

**Conclusion:**

NLGP’s binding to Dectin-1 receptors on murine dendritic cells, followed by the intracellular signal, lead to NFκB-mediated contrasting regulation of cytokine-transcriptions, initiating a pro-inflammatory immunopolarization, which amplifies further by the responding immune cells including T_c_ cells, alongside their enhanced cytotoxicity. These insights into the initiation of mammalian systemic immunomodulation by NLGP at cellular and molecular levels, may help uncovering its mode of action as a novel immunomodulator against human cancers, following clinical trials.

**Supplementary Information:**

The online version contains supplementary material available at 10.1186/s12964-024-01576-z.

## Introduction

Systemic ailments induce several glitches in the functionalities of the host immunity, favoring disease progression [1–3]. ‘Immunotherapeutic’ strategies, precisely designed to override these circumstances [4] have emerged as ‘remedies of choice’ to fight such diseases, including cancers. Consequently, immunomodulators are widely used to enhance or suppress specific immune-activities as required [5]. Beside several small molecule-based drugs (e.g. Thalidomide, Imiquimod etc.), plant-derived components (e.g. Curcumin, Resveratrol, Quercetin etc.) have been reported for their immunomodulatory antitumor activities [6–9]. In this queue, a 47 kDa water soluble glycoprotein, extracted from mature leaves of the plant ‘Neem’ (*Azadirachta indica*), named Neem leaf glycoprotein (NLGP) has shown promising attributes to overcome cancer-imposed immune-anergy. The ‘Neem’ tree belongs to the Meliaceae family of mahoganies and is endemic mostly to the tropical parts of Southeast Asia, including the Indian subcontinent. Regression of murine tumor growth (melanoma, sarcoma and carcinoma) have also been reported [10–12] due to the anticancer immunomodulatory actions of NLGP. Hence amongst all known glycoproteins, NLGP stands out as one having the ability to induce systemic immunomodulation in murine hosts, being a foreign (plant derived) compound.

Anti-tumor efficacy arises from the pro-inflammatory features of immunity, which constitute type 1 immune environment. NLGP prompts these features by enhancing the transcription factors, T-bet (*Tbx21*) [11, 13], Tbr2 (*Eomes*) [14] within T cells, decreasing GATA3 (*Gata3*) [11] simultaneously. Consequent hike in IFNγ production alongside enhanced cytotoxicity of effector T cells, induces pro-inflammatory cytokines, IL-2 and IL-12 from T cells and antigen presenting cells (APCs) [13] respectively. Suppression of GATA3 restricts secretion of anti-inflammatory cytokines, e.g., IL-4 from the T cells [13], thereby restraining type 2 immune components. Moreover, NLGP reduces the tumor-promoting phenotype M2, amongst tumor-associated macrophages (TAMs) as it downregulates the secretory (Ym1, FIZZ1 etc.) [15] and cell-surface (CD206, CD163 etc.) [16] M2 markers with a simultaneous increase of antitumor M1 markers (CD16, CD32 etc.) [17] in such cells. Elevated expressions of CD80, CD83 and CD86 mark NLGP-driven maturation of dendritic cells, which further aid in effective co-stimulatory interactions between the B7 molecules (CD80 and CD86) on APCs and CD28 on T cells, bringing about successful T cell activation [18]. However, participation of CTLA4, instead of CD28 hinders this process. Notable reduction of CTLA4 was observed among regulatory T cells (T_reg_) in vitro upon NLGP treatment as well [19]. NLGP drives the expansion of the cytotoxic T cells (T_c_) by RunX3 (*Runx3*) driven transcription of cytotoxicity-genes, while maintaining an overall helper-cytotoxic T cell (T_h_-T_c_) balance [20]. CD69 induction by NLGP suggests early activation of T_c_ cells [13] and constitutively upregulated Ki-67 and CD107a unfold their active proliferation and degranulation [11]. Overexpression of KLF2 (*Klf2*) by NLGP causes high *Sell* (CD62L) and *Ccr7* (CCR7) transcriptions in T_c_ cells, subsequently forming a stable central memory T_c_ cell population, ready for antigen re-encounter [21].

Multiple immunomodulatory functions of NLGP converge to the tumoricidal cytotoxicity of CD8^+^ T_c_ cells, which require APCs for activation. APCs therefore need to get influenced by NLGP first, to school those T cells optimally, enhancing the degranulation and cytotoxic secretions toward the tumor stroma. Thus, gateway of NLGP’s action that leads to immune-restoration of the host was explored in murine bone marrow-derived dendritic cells (mBMDCs) as professional antigen presenting cells (pAPCs) in vitro. NLGP being a glycoprotein, six glycoprotein receptors found on dendritic cells’ (DCs’) (and other pAPCs’) surfaces were studied and among them Dectin-1 (also known as CLEC7A) was found to be the putative receptor to recognize NLGP as its ligand. Subsequent studies revealed intracellular signal transduction through CARD9-aided NFκB activation, resulting in NLGP-induced alterations in the cytokine-secretion profile of DCs, which later manifests on to the production of multidirectional systemic effects of NLGP leading to regression of tumor growth [12] and metastasis [22].

## Methods

### Leaves of Neem (*Azadirachta indica*)

Mature leaves of the plant Neem (*Azadirachta indica*) were collected from a standard source (Salt Lake area of Kolkata, West Bengal, India), with permission of National Biodiversity Authority (NBA), an autonomous and statutory body of the Ministry of Environment, Forest and Climate change, Government of India (Ref. No. NBA/Tech Appl/9/518/12/14–15/4587). These leaves were used to prepare Neem Leaf Glycoprotein (NLGP), the ligand molecule under study.

### Cells

Hematopoietic stem cells were obtained from the marrow of the femur and tibia bones of C57BL/6 J mice (RRID:IMSR_JAX:000664), as primary cells, which were further cultured for 6 days to generate murine bone marrow-derived dendritic cells (mBMDCs).

B16-F10 (mouse melanoma) (RRID:CVCL_0159) from ATCC (Cat# CRL-6475) cells were cultured in Dulbecco’s Modified Eagle Medium (DMEM) with high glucose, supplemented with 10% fetal bovine serum (FBS) and 100 U/mL penicillin/streptomycin. Cultured cells were maintained in a 5% CO_2_ environment at 37 °C. Morphology of the cells were routinely monitored under microscope, but the cells were not further authenticated. The cell-free culture supernatant during 70%-100% confluency of B16-F10 was used to prepare the tumor conditioning media (TCM).

### Preparation of neem leaf glycoprotein

Neem (*Azadirachta indica*) leaves were thoroughly washed, shed-dried and pulverized to a powdered form. This was soaked in phosphate buffered saline (PBS) (pH 7.4) at 1% w/v ratio and was kept in continuous stirring motion overnight at 4 °C, for aqueous extraction of the leaf constituents. The slurry was centrifuged at 1500 rpm for 10 min at 4 °C, to collect the supernatant. This was membrane-filtered (0.22 µm) (Millipore, Darmstadt, Germany) to obtain the crude-aqueous extract, termed as neem leaf preparation (NLP). The endotoxin content of NLP was determined from different batches of freshly prepared NLP samples by Limulus Amebocyte Lysate (LAL) test, and the preparation having endotoxin content less than 6 pg/ml was used [10, 23].

The low-molecular weight solutes were removed from NLP, through extensive dialysis against PBS (pH 7.4) at 4 °C (4–5 times), using cellulose dialysis tubing membrane of 10 kDa molecular weight cut-off (MWCO). This was further concentrated by centrifugation at 4 °C, using the centricon system, Ultracel-10, Amicon Ultra-4 Centrifugal Filter Unit (Millipore Corporation, Bedford, MA, USA), which also has a 10 kDa MWCO. The biochemical identity of the resulting solution shows the properties of glycoprotein, as characterized earlier [24] and is designated as neem leaf glycoprotein (NLGP).

The purity of NLGP was checked by high-performance liquid chromatography (HPLC) through a µBondapak™ C18 prep column (pore size: 125 Å, particle size: 10 µm), under a pressure of 3 × 10^6^ N/m^2^, delivered by a Waters 501 HPLC pump, using acetonitrile–water as the solvent system of the mobile phase at a flow rate of 1 ml/min. Biological activity of purified NLGP was checked by tumor growth restriction assay before use. The protein concentration was determined by the method as described [25].

### Mass spectrometry of NLGP by LC–MS/MS and data processing

The protein-based backbone of NLGP was subjected to tandem mass spectrometric (LC–MS/MS) analysis and several NLGP-derived oligopeptides were identified. As NLGP yields a prominent peak near about 16th to 17th minute of retention time in HPLC, purity of NLGP sample was enriched by collecting the peak within that short time span. Following proteolytic digestion by trypsin (cleavage at the C terminus of Lys or Arg, unless followed by Pro) the oligopeptides generated from NLGP were subjected to MS/MS within Orbitrap Fusion Tribrid™ (Thermo Fisher Scientific, MA, USA). Following electrospray-based ionization (ESI) through EASY-Max NG™ (Thermo Fisher Scientific, MA, USA), the precursor ions (ionized oligopeptides) were run for MS1 (first mass spectrometric run of the precursor oligopeptide ions) through a quadrupole for HCD (High energy collision-induced dissociation) based fragmentation within the HCD cell. Since we wanted to identify an unknown sample from *Azadirachta indica*, all precursor ions from the survey scan were selected for fragmentation by HCD and data-independent acquisition (DIA). The fragment ion species (b- and y- ions) from each precursor ion were stabilized within the C-trap and were fed to the Orbitrap for MS2 (second mass spectrometric run of the fragment oligopeptide ions for detection). Due to their differential trajectories/orbits and speed within the Orbitrap, based on the corresponding m/z ratios, these fragment ions produced distinct peaks. The scan resolutions were set at 55,000 FWHM (full-width at half-maximum) at m/z 200 for MS1 and 17,500 FWHM at m/z 200 for MS2.

The primary spectrogram of all the precursor ions (oligopeptides from NLGP) after MS1 and the secondary spectrograms for each precursor ion (based on the m/z values of the corresponding fragment ions) after MS2, were determined in PEAKS Studio 11 (Bioinformatics Solutions Inc., Waterloo, Ontario, Canada). For peptide mass fingerprinting, the secondary spectrograms/spectra for each oligopeptide were read/sequenced by matching them with the existing profiles of proteins of different species, from the protein sequence database UniProtKB/Swiss-Prot, using PEAKS DB (PEAKS Studio 11, BSI, Ontario, Canada). Considering only monoisotopic masses (C12) of the precursor ions, their mass tolerances were set at 20.0 ppm and that of the fragment ions were set at 0.05 Da. The protease was selected as trypsin along with maximum missed cleavages as three. Carbamidomethylation was selected as fixed post translational modification (PTM) and oxidation of Methionine was considered as a variable PTM for this database search. Since, the sample protein (protein component of NLGP) was known to come from the green plant *Azadirachta indica* (Neem), the search was limited to the taxon viridiplantae of UniProtKB/Swiss-Prot, to avoid false positives from other taxa. Following database search, the PSM (peptide spectrum match) confidence/ the peptide score for each precursor ion was assigned as -10log_10_P, where the P-value is the probability that the match has occurred by chance and the oligopeptides with PSM higher than 58 were considered.

Further, to identify greater number of oligopeptides from NLGP, many more precursor ions were sequenced by de novo sequencing in PEAKS de novo (PEAKS Studio 11, BSI, Ontario, Canada). Instead of matching peptide profiles through database search, in de novo sequencing these precursor oligopeptide ions were read, based on their corresponding b- and y- series of fragment ions, where the mass tolerances of the precursor and the fragment ions were set at 15.0 ppm and 0.5 Da respectively and carbamidomethylation was considered as the fixed PTM. These oligopeptides were scored (De novo score) based on their average local confidence levels in percentage (ALC%) and peptides with very high ALC% (96 or more) were considered.

### Generation of mouse-bone marrow derived dendritic cells

Mouse bone marrow derived dendritic cells (mBMDCs) were generated by the method as described [26]. The femur and tibia bones were dissected out of C57BL/6 J mice aseptically and the marrow was flushed out within chilled PBS. After lysing the erythrocytes, the marrow cells were cultured within complete RPMI 1640 medium, supplemented with recombinant-mouse GM-CSF (rmGM-CSF) (10 ng/ml) and recombinant-mouse IL-4 (rmIL-4) (5 ng/ml). On day 3 the supplemented media was replenished and on day 6 the non-adherent cells were harvested from this culture as immature dendritic cells. For maturation, NLGP (1.5 µg/ml) was added separately to continue the culture for 48 h, whenever needed [18].

### Binding of NLGP-FITC with mouse-BMDCs

For Fluorescein isothiocyanate (FITC) tagging of NLGP, firstly a mixture of anhydrous Dimethylsulfoxide (DMSO) and triethylamine was added within a solution of NLGP. Three equivalents of FITC were further added to it, whilst stirring the solution and the reaction was continued for 12 more hrs at room temperature. After that, the residue was re-dissolved in DMSO (5 ml) and dialyzed against DMSO (MWCO: 3 kDa) to obtain the NLGP-FITC conjugate.

The mBMDCs were incubated with the FITC-coupled NLGP molecules (NLGP-FITC) (0.25 µl of NLGP-FITC added to 1 × 10^6^ cells/group) for 30 min at 4 °C in the dark. Later these cells were washed in PBS twice by centrifugation at 2000 rpm for 5 min each and were fixed by being resuspended in 1% paraformaldehyde solution. Interactions of NLGP with these mBMDCs were then studied by the localization of FITC-signal on/within these cells by fluorescence and confocal microscopy. Based on the fluorescent emission of FITC, comparative abundance of NLGP-binding to different groups of these cells was quantified by flow-cytometry as well.

### Fluorescence and confocal microscopy

With or without neutralization of the selected glycoprotein receptors in individual groups, the mBMDCs were incubated with NLGP-FITC (0.25 µl/10^6^ cells) at 4 °C for 30 min in the dark. After that, these cells were washed twice with PBS and were fixed with 1% paraformaldehyde (in PBS). Cells from each individual group were then mounted with Fluoroshield™-DAPI (Sigma). Images were captured by the widefield fluorescence microscope (Leica, BM 4000B, Germany) and the confocal microscope (Carl Zeiss, 63X/1.4 oil DIC Plan-APOCHROMAT objective, Germany). NLGP’s binding and internalization within these DCs were visualized by the localized fluorescent-emission of FITC from the surface as well as the interior of the cells. The extent of fluorescent-signal-intensities from individual cells was quantified by Fiji (a distribution of ImageJ) in different fields. The overall fluorescence emission coming out of a single cell was determined as its corrected total cell fluorescence (CTCF), measured as:

CTCF = Integrated Density – (Area of the cell × Mean grey value of its adjacent background).

MFI of each field was determined as the mean of the CTCFs of all the cells present within the field. To distinguish the intracellular pool of NLGP-FITC from the surface-bound, the interior distribution of the fluorescence was visualized within a 3D view of a few cells, which were incubated with NLGP-FITC for 1 h at 4 °C, prior to washing and fixation. Forty-two consecutive 2D images of the same field (different slices focused on individual consecutive layers along a specific interval of cell-thickness through the Z-axis) captured in the confocal microscope, were stacked together. This Z-stack was further processed through the ‘3D viewer’ plugin of Fiji/ImageJ2 to form 3D views (surface view, volume view and superimposition of both/composite view) and Z-projections (based on sum slices and maximum intensity respectively) of the field. Longitudinal sections, exposing the cell-interiors through these 3D views (volume view and composite view) were obtained and the views were rotated around the Y-axis or the vertical axis clockwise and were further magnified to have a clear observation of the pool of NLGP-FITC, within the cell-interiors.

### Tumor conditioning of mouse-BMDCs

B16-F10 melanoma cells were cultured up to 70% confluency within complete DMEM high glucose cell culture media, containing 10% (v/v) heat-inactivated FBS, 2 mM L-glutamine and penstrep (50 units/ml Penicillin; 50 µg/ml Streptomycin) at 37 °C in 5% CO_2_. At the time-point of 70% cell-confluency, the culture media was replenished completely. On reaching 100% confluency, the culture media containing the tumor-cell secretions was collected, centrifuged at 2000 rpm for 5 min and membrane-filtered (0.22 µm) (Millipore, Darmstadt, Germany) to prepare the tumor conditioning media (TCM).

For tumor conditioning, the TCM was added to complete RPMI 1640 medium containing 10% (v/v) heat-inactivated FBS, 2 mM L-glutamine and penstrep (50 units/ml Penicillin; 50 µg/ml Streptomycin) at 1/4th ratio. Subsequently, the mBMDCs were cultured within this media (1 × 10^6^ cells/ml) for 48 h at 37 °C with 5% CO_2_, for these cells to acquire the tumor-induced phenotypes.

### Neutralization of cell surface glycoprotein receptors

Six glycoprotein receptors present on mBMDC surfaces, namely, Mannose binding receptor, Dectin-1, Dectin-2, DC-SIGN, DEC-205 and DNGR1 were neutralized. Mannan (10 µM) (Sigma-Aldrich, St. Louis, Missouri, USA), obtained from *Saccharomyces cerevisiae* and Laminarin (10 µM) (InvivoGen, San Diego, CA, USA), prepared from *Laminaria digitata* were used to block MBRs and Dectin-1 receptors respectively. Neutralizing antibodies (5 µg/ml) were used to block the other four receptor types. After addition of the corresponding inhibitors, each of the groups of cells was incubated within complete RPMI-1640 medium, for one hr at 37 °C, supplied with 5% CO_2_. The receptor-neutralizations were done prior to the addition of NLGP or NLGP-FITC to the mBMDCs.

### Flow cytometry

The extent of NLGP-FITC binding to mBMDCs was analyzed by flow cytometry. The cells were incubated with NLGP-FITC (0.25 µl/10^6^ cells) for 30 min in dark followed by PBS washing. After fixing the cells in 1% paraformaldehyde, binding of NLGP-FITC was compared among different groups on neutralizing different receptors. To quantify cytokine (IL-10 and IL-12p35) production rates by tumor conditioned mBMDCs after receptor-neutralization and NLGP treatment, the cells were treated with 10 nM of Brefeldin A for 2 h for partial intracellular arrest of cytokines. Followed by membrane-permeabilization by Cytofix/Cytoperm reagents according to the manufacturers protocol (BD Biosciences, San Diego, CA), and incubations with corresponding fluorochrome-tagged antibodies, cytokine production rates were compared among separate cell groups by flow cytometry.

For each experiment, flow cytometry was performed as described [27]. In brief, cytofluorimetric data was generated by acquisition of 10,000 events per group on a FACSCalibur flow-cytometer (Becton Dickinson, Mountainview, CA) and background autofluorescence was subtracted by negative isotype controls. Further, percentage of each positive population was determined using region statistics by FlowJo v10.7.2 (Tree Star, Ashland, OR) and the representations were made as per convenience of explanation.

### ELISA

ELISA was performed to quantify cytokine-secretions (IL-10 and IL-12p35) from mBMDCs and to detect β-Glucans’ presence in NLGP. Cytokines harvested within 48-h culture supernatants of mBMDCs, were immobilized by overnight incubation, within individual wells (50 µl/well) of a 96-well microtiter plate at 4 °C, from different experimental groups. Whereas, to address the possibility of β-Glucans’ presence in NLGP, serially diluted (twofold) NLGP-samples were immobilized within a 96-well microtiter plate, in the same way. In both cases, the wells were blocked with 5% BSA (200 µl/well) for 3 h at 4 °C, followed by labelling with corresponding primary antibodies (1:500 in 1% BSA) by overnight incubation (50 µl/well) at 4 °C. The wells were washed thrice with PBS-Tween 20 (200 µl/well), followed by incubation (2 h in dark) with HRP-conjugated secondary antibody (1:1000 in 1% BSA) (50 µl/well). Addition of TMB substrate (BD OptEIA, BD Biosciences) initiated a reaction in these wells, producing a visible colored-product (light-blue/cyan). This color was changed to yellow as the reaction was terminated with 1 (N) H_2_SO_4_ and the optical densities of different groups were quantified at 450 nm by spectrophotometry using the software, SoftMax Pro 7.1, in Spectramax i3x (Molecular Devices, San Jose, USA).

### cDNA preparation, reverse-transcriptase PCR and qPCR

Quantitative and semi-quantitative PCRs were performed from the RNA extracted from mBMDCs as described [27, 28]. Total RNA was extracted from mBMDCs by phase-separation method using Trizol reagent (Invitrogen, Camarillo, CA), and reverse transcription was performed to prepare cDNA using RevertAid First Strand cDNA Synthesis Kit (Thermo Fisher Scientific, MA, USA). Intended gene-segments were amplified by PCR, using 2X Go Taq Green Mix (Promega, WI, USA), in C1000 Touch™ thermal cycler (Bio-Rad, USA) and the products were electrophoresed in 1.5% agarose gels. The gels were stained with Ethidium Bromide and resulting band images were captured by ChemiDoc XRS + (BioRad Laboratories, CA, USA). Band-intensities were then quantified using Image Lab 6.1.

Quantitative PCR of the gene-segments were performed using Takyon™ Low ROX SYBR 2X MasterMix blue dTTP (Eurogentec, Europe) in the Applied Biosystems™ 7500 Real-Time PCR System (ThermoFisher Scientific, MA, USA). Fold change of mRNA expression of each experimental group compared to the control group, was quantified as 2^−ΔΔCt^, derived from its C_t_ (cycle threshold) value. β-actin was kept as an internal control gene. Gene-specific primers are tabulated in Supplementary Table 6.

### Post-transcriptional suppression of Dectin-1 by RNAi

Expression of Dectin-1 was knocked down in vitro*,* among murine BMDCs by post transcriptional gene silencing (PTGS) using RNA interference (RNAi). An siRNA molecule was constructed using the Ambion Silencer® siRNA Construction Kit (Life Technologies, USA). A pair of 29-mer ssDNA-oligonucleotides was taken for the synthesis of each siRNA strand (Supplementary Table 6). Individual strands of the siRNA were then transcribed, using the T7 promoter, hybridized and further cleaved, as described in the manufacturer’s protocol. Accordingly, a double stranded 21-mer siRNA with 3’ Uridine-dimers as overhangs in both strands was synthesized for Dectin-1 knockdown (5’-GGCCCAGGGGAUCAGAGAAUU-3’ as the guide strand and 5’-UUCUCUGAUCCCCUGGGCCUU-3’ as the passenger strand). Following quantification by Nanodrop Spectrophotometer (MicroDigital Co., Ltd. Korea), this siRNA was introduced within mBMDCs by transfection, using the Lipofectamine® 2000 Transfection Reagent (Thermo Fisher Scientific, MA, USA) and serum-reduced Opti-MEM® medium (Thermo Fisher Scientific, MA, USA). The siRNA (50 µM) and Lipofectamine (6 µl) were separately added to two Opti-MEM aliquots (250 µl each) and both were kept at room temperature for 5 min. Then, both were mixed, up to a final volume of 500 µl and kept at room temperature for 20 min. This mixture was added in vitro to 2-h serum-deprived, 70% confluent culture of mBMDCs, so that the final concentration of the siRNA becomes 50 nM within the media. A non-specific siRNA was parallelly used as control. Expression of Dectin-1 was quantified in different groups of cells by western botting, to check the extent of downregulation. The mBMDCs with suppressed levels of Dectin-1 were further checked by microscopy, PCR, flow-cytometry and ELISA for NLGP-binding and consequent cytokine-release patterns, compared to controls.

### Preparation of cytosolic, nuclear and total protein lysates from mBMDCs

Cytosolic, nuclear as well as total cellular protein contents were extracted from mBMDCs as described [21, 27]. In short, the mBMDCs were suspended within ice-cold ‘cytosolic protein extraction buffer’ and was incubated for 1 h at 4 °C. Following centrifugation of these cells at 6000 rpm for 5 min, the supernatant was collected as the ‘cytosolic protein lysate’. The pellet was resuspended within ‘nuclear protein extraction buffer’ and was vortexed for 30 min at 4 °C. The resulting suspension was centrifuged at 12,000 rpm for 10 min to collect the supernatant as the ‘nuclear protein lysate’ [21].

To extract the total cellular protein content, the cells were suspended within a protein lysis buffer (1% Triton X-100, 10 mmol/l Tris–HCl [pH 7.4], 1 mM EDTA, 0.2 mM sodium orthovanadate, 0.5% Nonidet P-40 in PBS, alongwith a protease inhibitor mix). Following an incubation of 30 min at 4 °C for cell lysis, the lysate was centrifuged at 12,000 rpm at 4 °C, for 30 min. The supernatant was collected as the ‘total protein lysate’ [27].

### Western blotting

Western blotting was performed as described [27]. Cellular protein lysates, mixed with Laemmli buffer (50 µg/ml) were boiled for 5 min, fractionated by SDS-PAGE (10–12% gels), transferred onto polyvinylidene difluoride (PVDF) membranes (Advansta, CA, USA) followed by blocking with 5–8% BSA for 2 h. Upon overnight incubation with corresponding primary antibodies, the bands on the membranes were probed with specific HRP-conjugated secondary antibodies for 2 h. These bands were visualized by chemiluminescence, using WesternBright ECL HRP substrate (Advansta, CA, USA) and were captured within ChemiDoc XRS + (BioRad Laboratories, CA, USA). Band intensities were quantified in Image Lab 6.1.

### Co-immunoprecipitation

The association of CARD9 with Bcl10 and MALT1 was checked by co-immunoprecipitation assay, as described [29]. Mouse BMDCs were lysed within RIPA buffer and 50 µg of lysates were pre-cleared within 50% (w/v) protein G-Sepharose beads at 4 °C for 1 h and centrifuged at 20,000 g at 4 °C for 10 min. The supernatant was incubated and mixed overnight with anti-CARD9 antibody at 4 °C. Resulting immune complexes were incubated with Protein G-Sepharose beads for 2 h at 4 °C. These complexes were boiled with Laemmli buffer for 5 min to separate out the constituent proteins and followed by SDS-PAGE, the presence/absence of Bcl10 and MALT1 was checked by western blotting.

### Chromatin-immunoprecipitation assay

After nuclear translocation of NFκB, whether the consequent NFκB dimers consisting of p65 (from *Rela* gene) and p50 (form *Nfkb1* gene), modulate *Il10* and *Il12a* gene transcription rates was checked by performing chromatin immunoprecipitation (ChIP) assay. Two thousands of nucleotides from the gene sequences (keeping the transcription start sites in the middles) of both *Il10* and *Il12a* of the house mouse (*Mus musculus*) were found in the eukaryotic promoter database (EPD), and single nucleotides, marking the specific positions of the transcription factor binding motifs (TFBMs) for binding of p65 and p50, were identified within them (-856th nucleotide for p50-binding and -484th nucleotide for p65-binding to *Il10* as well as -794th nucleotide for p65-binding and -512th nucleotide for p50-binding to *Il12a* genes respectively) using JASPAR CORE 2018 vertebrates, as the database for transcription factor binding sites. The consensus sequences for binding of mouse-p65 and p50 were obtained from the commercial version of TRANScription FACtor database (TRANSFAC 8.3), applying the matrix-based TFBM-prediction algorithm named PROMO in the ALGGEN (BarcelonaTech) web server. Sequences of short DNA stretches that contain the TFBMs were aligned with the corresponding TFBM-consensus sequences in the sequence alignment editor, BioEdit 7.2.5 (NCSU, North Carolina, USA), to locate the exact TFBM regions in those genes (-860th to -849th nucleotides for p50-binding and -488th to -479th nucleotides for p65 binding in *Il10* gene sequence, along with -798th to -788th nucleotides for p65-binding and -517th to -505th nucleotides for p50-binding in the *Il12a* gene). To address the probable binding of different dimeric combinations of NFκB to the TFBMs of *Il10* and *Il12a* genes, individual monomer-binding (for both p65 and p50) was checked at the corresponding sites. The transient DNA–protein complexes were stabilized by crosslinking, as the mBMDCs (1 × 10^6^ cells/group) were treated with 1% formaldehyde for 10 min at 37 °C. These cells were washed twice with chilled PBS (pH 7.4, containing 1 mM PMSF and 1 µg/ml pepstatin A as protease inhibitors) and lysed within SDS Lysis Buffer (containing protease inhibitors) by a 10 min’ incubation on ice. The DNA content cross-linked to bound proteins, present within the resulting lysate was then sheared to small (~ 200–1000 bp) DNA stretches by sonication (5 pulses of 30%, 5 s each in UP400S, Ultrasonicator, Hielscher, NJ, USA). The lysate was then pre-cleared using Salmon Sperm DNA/Protein A Agarose-50% Slurry by incubation for 30 min at 4 °C with agitation, followed by brief centrifugation to spin down the beads. A part of the supernatant, containing all protein-DNA complexes was collected out of the sample as input DNA sample (positive control for ChIP), before immunoprecipitation. The desired protein-DNA complexes were immunoprecipitated with antibodies against p65 and p50 (5 µg each) by overnight incubation at 4 °C with rotation. These antibody-protein-DNA complexes were captured by Protein A-agarose beads, present within Salmon Sperm DNA/Protein A Agarose-50% Slurry by incubation for 1 h at 4 °C with rotation. These beads along with the immobilized complexes were washed in appropriate buffers and the DNA–protein complexes were eluted out within elution buffer, by 15 min’ incubation at room temperature. DNA–protein crosslinks were reversed by 5 M NaCl at 65 °C for 4 h, and the proteins were degraded by Proteinase K treatment (with 0.5 M EDTA and 1 M Tris–HCl) at 45 °C for 1 h, leaving only the DNA within the eluates. From these eluates, DNA was extracted using phenol/ chloroform and precipitated with 70% ethanol. Presence/absence of the corresponding TFBMs (for both p65 and p50 binding near both *Il10* and *Il12a* gene promoters) among these DNA were verified by PCR-based amplification of those sites, using appropriate primer-sets, mentioned in Supplementary Table 6.

### In silico processing of molecular structures for docking studies

The extended β-Glucan chains present on the surface of the ligands of Dectin-1 (CLEC7A) receptors, adhere to these receptors to bring about the overall receptor-ligand binding. Thus, in an attempt to understand the molecular mechanisms of NLGP-Dectin-1 binding in the best possible way, favorable associations of Dectin-1 and β-Glucans were elucidated in terms of thermodynamic stability in silico, by molecular docking studies. Two different β-Glucan chains (CHEBI:133,802 and CHEBI:18,246) were procured from the online dictionary ‘Chemical Entities of Biological Interest’ (ChEBI) of EMBL-EBI as Molfiles (.mol), which were converted to the partially charged file format (.pdbqt) by Open Babel GUI (Open Babel: The Open Source Chemistry Toolbox). Three common β-Glucan structures namely, β-_D_-Glc-(1 → 3)-[β-_D_-Glc-(1 → 6)]-β-_D_-Glc-(1 → 3)-β-_D_-Glc, β-_D_-Glc-(1 → 3)-β-_D_-Glc-(1 → 3)-β-_D_-Glc-(1 → 3)-β-_D_-Glc and β-_D_-Glc-(1 → 4)-β-_D_-Glc-(1 → 4)-β-_D_-Glc were prepared as small molecule-ligands from these two structures in BIOVIA Discovery Studio Visualizer (Dassault Systemes, Île-de-France, France). The quaternary dimeric crystal structure of murine Dectin-1 (2BPE) was obtained from RCSB Protein Data Bank. This crystal structure of Dectin-1 was refined by removing the embedded water molecules and the excess hetero atoms, followed by repairing the missing atoms of the amino acid residues and polar hydrogens and addition and uniform distribution of Kollman charges, in AutoDock 4.2.6 (Scripps Research, La Jolla, CA, USA).

### Molecular docking

Centre of the active site of Dectin-1 was determined from the X, Y and Z coordinates of the active site amino acids, obtained from the.pdb file of the crystal structure. A spatial grid-box was generated using Autogrid 4.2.6 around the centre, taking 80 points in each dimension with 0.5 Å spacing. Dockings were performed according to the Genetic algorithm with 30 runs for each ligand in AutoDock 4.2.6 (Scripps Research, La Jolla, CA, USA). The Lamarckian GA (4.2) outcomes of docked complexes were analyzed and the ones with the lowest binding free energy values were taken (with.pdbqt extensions) as the most stable conformations for each ligand-receptor docked complex. The individual root mean square deviations (RMSDs), binding energies (Kcal/mol) and inhibition constants (K_i_) for each ligand (with Dectin-1) were found in the respective Docking log files and the numbers of generated H-bonds along with the amino acids of Dectin-1, involved in each interaction was obtained using BIOVIA Discovery Studio Visualizer (Dassault Systemes, Île-de-France, France). All diagrams, representing the molecular interactions were prepared in BIOVIA Discovery Studio Visualizer.

### Statistical analyses

Each set of experimental data represent the ‘Mean ± SD’ of independent replicates. The exact numbers of experimental replicates (n values) are mentioned in the corresponding figure legends. Gaussian/Normal distribution of the data was checked by Shapiro–Wilk test or Kolmogorov–Smirnov test or D'Agostino & Pearson test (α = 0.05) (as applicable), prior to statistical significance determination. Statistical significance was determined by Two-tailed unpaired t test with Welch’s correction (for 2 groups) and One-way ANOVA, followed by Tukey’s multiple comparisons test (for more than 2 groups) for ungrouped data. For grouped data, Two-way ANOVA (more than 2 groups) was used, followed by Tukey’s multiple comparisons test. The F values for one-way ANOVA and t values for the t test as well as the degrees of freedom for ANOVA and t tests are mentioned in the corresponding figure legends. The difference between two experimental groups, attaining p ≤ 0.05 was considered to be significant. Both significant and non-significant P values are mentioned in the figures. Linear regression was performed to measure a dependent variable, based on the regression equation, in a ‘part-to-whole’ analysis (quantification of β-Glucan within NLGP). Initial tabulation of data was done in MS-Excel, and all statistical analyses were performed in Prism 8.4.2 (GraphPad, San Diego, CA, USA).

## Results

### A water-soluble component of mature leaves from Azadirachta indica consists of sugar-modules with a polypeptide-based foundation

**Fig. 1:**
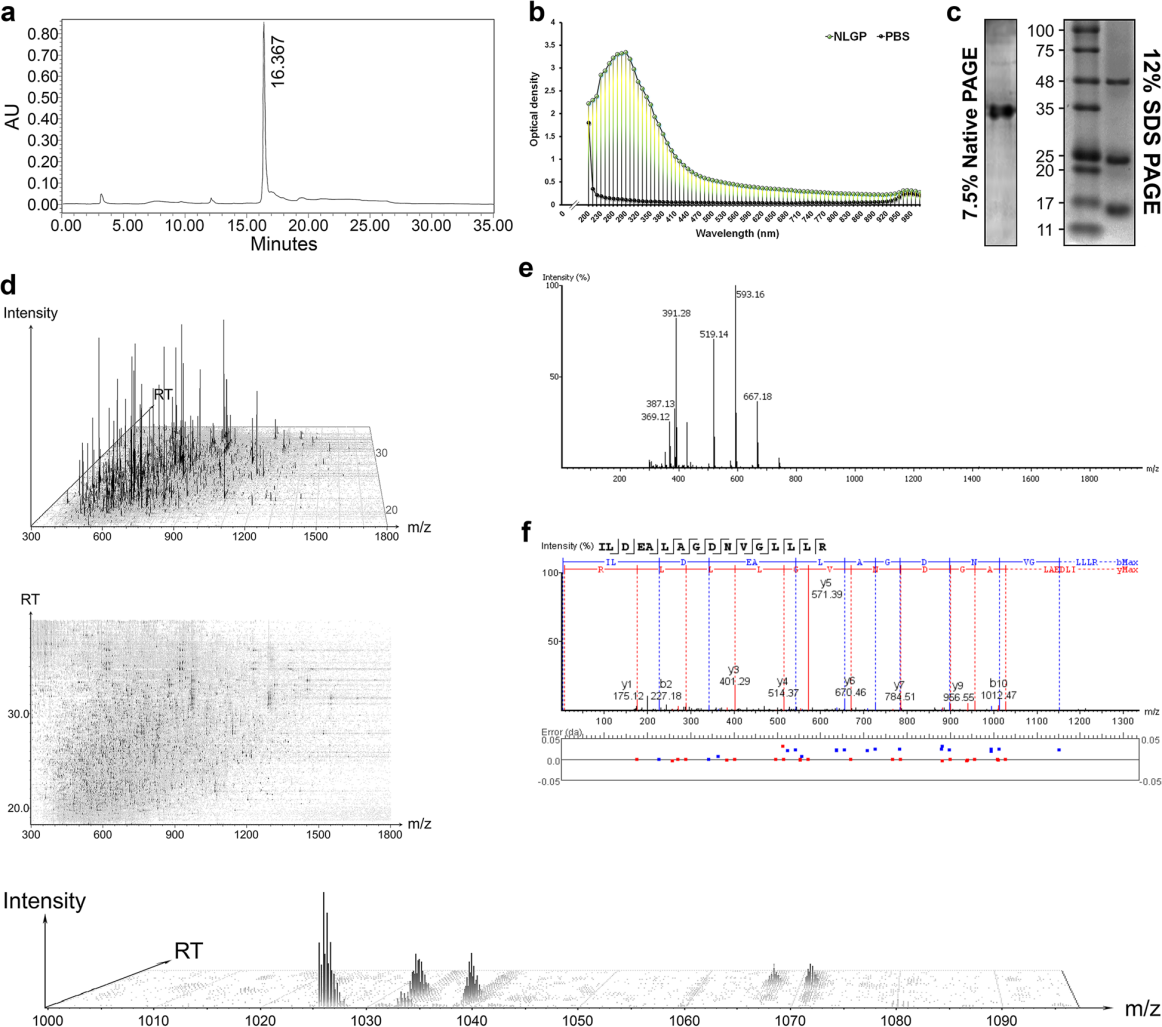
The water-soluble component of *Azadirachta indica*, called Neem leaf glycoprotein or NLGP has a polypeptide-based foundation. **a** Chromatogram of NLGP from high-performance liquid chromatography (AU stands for absorbance units, in 210 nm). **b** Absorbance spectrum of NLGP from wavelengths of 200 nm to 1000 nm, compared to that of phosphate buffered saline. **c** Electrophoresis of NLGP in polyacrylamide gels. **d** LC/MS views of the fragment ion hits after MS2 of tandem mass spectrometry of the protein-moiety of NLGP, with (upper) and without (middle) relative intensities. Formation of fragment ion-peaks in a magnified LC/MS view (lower) (RT stands for retention time, m/z is the mass to charge ratio). **e** Primary mass spectrogram of precursor ions (NLGP-peptides) after MS1 through data independent acquisition. **f** Deduction of a peptide spectrum (secondary mass spectrogram) from b- and y- fragment ion-hits (da stands for daltons)

Biochemical characteristics of the neem (*Azadirachta indica*) leaf-ingredient under study unveiled its identity as a glycoprotein, hence it was termed as Neem Leaf Glycoprotein (NLGP). Following, aqueous extraction, dialysis-based filtration and enrichment by centrifugal ultrafiltration, the extent of NLGP’s purification was checked by high-performance liquid chromatography (HPLC) through a µBondapak™ C18 Prep Column using acetonitrile–water as the mobile phase, at a flow rate of 1 ml/min under 3 × 10^6^ N/m^2^ pressure. This yielded a sharp peak at 16.367th minute indicating elution of nearly the whole analyte at that point of time from stationary phase (Fig. 1a). Thus, purity of this neem-derived glycoprotein was ensured. NLGP has shown highest optical densities around the wavelength of 280 nm (Fig. 1b) within a spectrum of 200 to 1000 nm, confirming protein to be its major constituent. On being electrophoresed through 7.5% Native PAGE (polyacrylamide gel electrophoresis), it migrated like an intact protein yielding a single band upon staining. However, in SDS PAGE, this leaf-ingredient split up in three distinct bands (Fig. 1c). This suggested the presence of three polypeptide chains to be attached and folded into a quaternary protein structure as the backbone of NLGP. Being unfolded by Sodium dodecyl sulfate (SDS), and upon the reduction of intra- and/or inter-molecular disulfide bridges, these three polypeptides get separated in SDS PAGE. As these three polypeptides originally agglomerate to form the quaternary structure of the protein-moiety of NLGP, weak molecular interactions including disulfide bonds, Van der Waals forces etc. would govern the adherence points within their primary structures. Secondary structures like the helices, sheets, loops and other possible supersecondary elements would direct further appropriate folding. Since, all these elements contribute precisely to form the functional motifs and domains, any irregularity in any of these would lead to local or overall compromisation of the functionalities of this molecule. The three constituent polypeptide chains are thus indispensable in the exact manner that they contribute to the molecular structure of NLGP. Presence of carbohydrate residues in this neem leaf-component was suggested by staining gels with the periodic acid-Schiff reagent. Furthermore, trimming off NLGP’s sugar-moiety with neuraminidase, abolished its previously known anti-tumor activities. Though being the less abundant components within the overall structure, these glycans are thought to carry out the key regulatory functions of NLGP. Dectin-1-binding is solely dependent on peripheral β-D-Glucan chains of NLGP as described later. Some other peripheral carbohydrates may participate in increasing the overall solubility. Some saccharide residues may also be locally embedded, depending on the mutual sugar–protein conformation. In a parallel study the N-glycans present within NLGP are being collectively characterized through mass spectrometry, which would provide further detailed insights on these sugar-structures, leading to more precise understandings to their functions.

Composition of the protein-based backbone of NLGP was elucidated by thioglycolic acid-based hydrolysis, followed by column chromatography-based amino acid residue detection (Table 1). Further, this protein portion was subjected to tandem mass spectrometry (LC–MS/MS) (described in Methods), generating a primary mass spectrogram with precursor ion-peaks (Fig. 1e) and many secondary spectrograms (from fragment ions) (as in Fig. 1f). LC/MS (liquid chromatography followed by mass spectrometry) views segregate the fragment ions based on mass-to-charge ratio, over a retention time window with significant ion-hits (Fig. 1d). Through peptide mass fingerprinting, oligopeptides were identified by matches with proteins from taxon viridiplantae, within UniProtKB/SwissProt (Table S1). Several other oligopeptides were sequenced from the precursor ions by de novo sequencing (Table S2).

**Table 1:** Amino acid composition of the protein-moiety of NLGP

Amino acid(s)	Abbreviations		Molecular weight	Average micrograms from raw data	Molar percentage composition
Asparagine and Aspartic acid	Asx	B	115.1	10.288	11.4%
Glutamine or Glutamic acid	Glx	Z	129.1	13.530	13.3%
Serine	Ser	S	87.1	5.893	8.6%
Histidine	His	H	137.2	1.244	1.2%
Glycine	Gly	G	57.1	4.399	9.8%
Threonine	Thr	T	101.1	5.068	6.4%
Alanine	Ala	A	71.1	5.703	10.2%
Arginine	Arg	R	156.2	5.208	4.2%
Tyrosine	Tyr	Y	163.2	4.035	3.1%
Valine	Val	V	99.1	4.271	5.5%
Methionine	Met	M	131.2	1.434	1.4%
Phenylalanine	Phe	F	147.2	4.505	3.9%
Isoleucine	Ile	I	113.2	3.847	4.3%
Leucine	Leu	L	113.2	6.545	7.3%
Lysine	Lys	K	128.2	3.782	3.7%
Proline	Pro	P	97.1	4.330	5.7%

### NLGP binds to the C-type lectin receptor Dectin-1 on dendritic cell surface

**Fig. 2:**
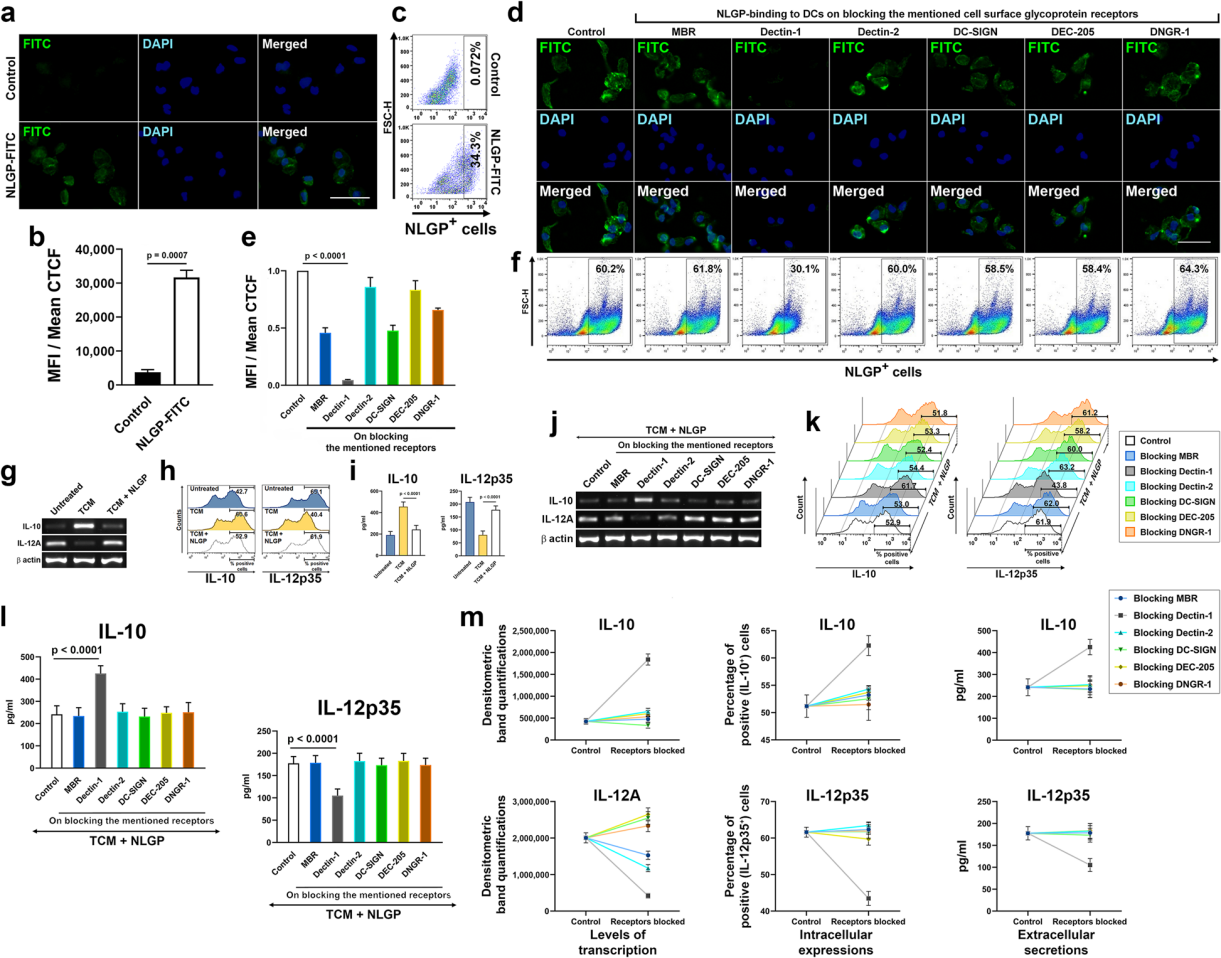
NLGP binds to dendritic cells through Dectin-1 receptors. **a** Widefield fluorescence micrographs of NLGP-FITC bound mouse BMDCs, compared to control (scale bar: 20 µm). **b** MFIs of experimental replicates (*n* = 3) of such cell groups as mean ± SD (MFI and CTCF denotes mean fluorescence intensity and corrected total cell fluorescence respectively). **c** Flow cytometric quantifications of two such representative groups. **d** Widefield fluorescence micrographs of NLGP-FITC-binding to mBMDCs on neutralizing mentioned cell-surface C-type lectins (scale bar: 20 µm) (MBR denotes mannose binding receptors). **e** MFIs of experimental replicates (*n* = 3) of such groups as mean ± SD (TCM denotes tumor conditioning media). **f** Flow cytometric analyses of these groups. **g** Agarose gel electrophoresis for reverse transcription-based semi quantitative PCR of IL-10 and IL-12A or IL-12p35 transcripts from control, TCM and TCM + NLGP treated mBMDC groups. **h** Flow cytometric quantifications of intracellular IL-10 and IL-12p35 from previous groups as offset histograms. **i** Secretion of IL-10 and IL-12p35 within 48-h culture supernatants of previous groups measured by ELISA, from experimental replicates (*n* = 6) as mean ± SD. **j** Agarose gel electrophoresis after reverse transcription-based semi quantitative PCR, measuring IL-10 and IL-12A or IL-12p35 transcripts on neutralizing the mentioned cell-surface C-type lectins on separate groups of TCM + NLGP treated mBMDCs. **k** Flow cytometric quantifications of intracellular IL-10 and IL-12p35 from receptor-neutralized groups as staggered-offset histograms. **l** Secretion of IL-10 and IL-12p35, quantified within 48-h culture supernatant of these groups (*n* = 6) by ELISA as mean ± SD. **m** Effects of receptor-neutralizations on IL-10 and IL-12A or IL-12p35 production, quantified as transcripts, intracellular proteins, and secreted proteins, compared to control, collectively represented through line diagrams as mean ± SD from (**j**), (**k**) and (**l**). β-actin was taken as loading control in gels. Numerical data of (**b**), were tested by two-tailed unpaired T test with Welch’s correction (*t* = 21.52, df = 2.488) and those of (**e**), (**i**) and (**l**) were tested by one-way ANOVA (*F* = 123.5; 79.20, 96.86; 21.27, 18.09 respectively, df = 20; 17, 17; 41, 41 respectively) followed by Tukey’s multiple comparisons test. Normal distribution of the data were verified before tests (See also Fig. S1)

Activation of CD8^+^ T cells is a prime attribute of NLGP’s anti-tumor activity. Effector functions of these T cells are driven by antigen presentation, co-stimulatory interactions and appropriate cytokine combinations from APCs. Since the APCs deliver the anticancer immune-thrust imposed by NLGP to cytotoxic effector CD8^+^ T cells, direct involvement of the APCs seemed highly possible in initiating NLGP’s systemic effects. Thus, dendritic cells were studied as the major pAPCs and any direct interaction of NLGP to these cells was checked by incubating mBMDCs with Fluorescin isothiocyanate (FITC)-coupled NLGP, followed by washing and fixation. Nuclei of the cells were stained with 4′,6-diamidino-2-phenylindole (DAPI). Fluorescence micrographs displayed prominent binding of NLGP to the mBMDCs (Fig. 2a, b) as the mean fluorescence intensity (MFI) (calculated as mean of the corrected total cell fluorescence or mean CTCF values from experimental replicates) of the FITC from these cells were 8.5 times of that of the control cells (mBMDCs, incubated with anti-Rat IgG-FITC, washed and fixed) (Table S3). Flow-cytometric analyses were also performed on these groups of cells, where irrespective of size and granularity, the whole dendritic cell populations were considered for doublet corrections, followed by subtraction of autofluorescence through negative isotype controls. The percentages of FITC-positive cells were then determined for each group using region statistics along a logarithmic scale on dot-plots, having a pseudocolor-gradient. Prominent binding of NLGP-FITC to the mBMDCs were also found by these flow cytometric studies (Fig. 2c). Such evidences of NLGP-DC interactions led us to explore the appropriate membrane receptor involved in NLGP binding.

As a glycoprotein, NLGP would attach to a glycoprotein-binding receptor. Hence, six glycoprotein-binding immunoreceptors (C-type lectin receptors) present on DC surfaces were screened to look for a probable candidate that can bind NLGP. These receptors were Mannose receptor (also known as Mannose-binding receptor or MBR or CD206), Dectin-1 (also known as CLEC7A or CD369), Dectin-2 (also known as CLEC6A), DC-SIGN (also known as CD209), DEC-205 (also known as CD205) and DNGR-1 (also known as CLEC9A or CD370). Each of these receptor types was blocked on separate groups of mouse-bone marrow derived dendritic cells (BMDCs) by corresponding antagonistic ligands or neutralizing antibodies and these groups of cells were incubated with NLGP-FITC. Binding of NLGP-FITC dropped down significantly on blocking Dectin-1 receptors by the antagonist Laminarin on DC surfaces as per the MFIs from fluorescence micrographs. Such decline in NLGP-DC interactions was absent, when the other five receptors were blocked (Fig. 2d, e; Table S4). Flow cytometric analyses revealed that nearly 30% of cells lost their inherent affinity towards NLGP on obstructing Dectin-1 receptors, compared to the control and the other groups (Fig. 2f). These observations strongly indicate a loss in binding of NLGP to the overall dendritic cell population to a huge extent on obstructing the Dectin-1 receptors, followed by its effects on subsequent interactions and corresponding functionalities.

**Fig. 3:**
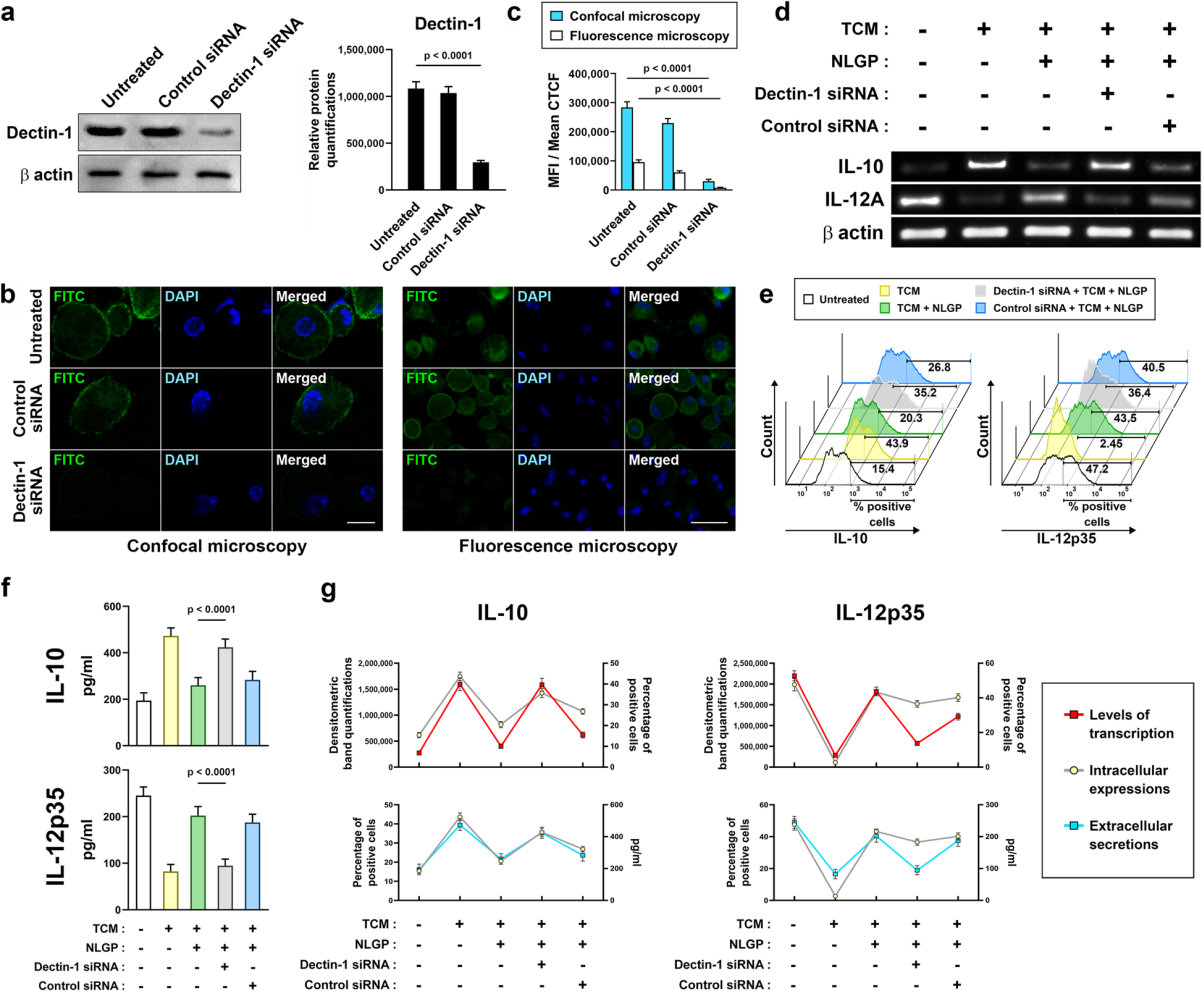
Post-transcriptional silencing of Dectin-1 gene expression on mBMDCs brings down NLGP binding to the cells with functional consequences at cytokine production levels. **a** Western blotting to measure Dectin-1 protein suppression by RNAi in mBMDCs, compared to controls (left) (*n* = 3), as mean ± SD (right). **b** Extent of NLGP-FITC binding to mBMDCs on silencing Dectin-1 compared to controls, in confocal (left) (scale bar: 10 µm) and widefield (right) (scale bar: 20 µm) fluorescence micrographs. **c** MFIs from replicates of those fluorescence microscopy-based observations (*n* = 3), as mean ± SD (MFI and CTCF stand for mean fluorescence intensity and corrected total cell fluorescence respectively). **d** Agarose gel electrophoresis, quantifying IL-10 and IL-12A or IL-12p35 transcripts by RT-PCR, to assess effects of Dectin-1 depletion by RNAi vs control siRNA, on TCM and NLGP’s gradual influence on mBMDCs (TCM stands for tumor conditioning media). **e** Flow cytometric quantifications of intracellular IL-10 and IL-12p35 of same groups as staggered-offset histograms. **f** IL-10 and IL-12p35 secretions measured within 48-h culture supernatants of the same groups (*n* = 6) by ELISA, represented as mean ± SD. **g** Trend of cytokine production from the same mBMDC groups, through line diagrams, as transcription (red line) vs. intracellular expression (grey line) (above) and intracellular expression (grey line) vs. secretion (cyan/blue line) (below) for both IL-10 (left) and IL-12A or IL-12p35 (right) as mean ± SD from (**d**), (**e**) and (**f**). β-actin was taken as loading control in blots and gels. Numerical data of (**a**) were tested by one-way ANOVA (*F* = 169.1, df = 8), and those of (**c**) were tested by two-way ANOVA, both followed by Tukey’s multiple comparisons test. Numerical data of (**f**) were tested by one-way ANOVA (*F* = 67.74 and 102.9 respectively, df = 29 and 29 respectively), followed by Tukey’s multiple comparisons test. Gaussian distribution of data were verified before each test (See also Fig. S2a-d)

Though, the Dectin-1 receptors were found to bind NLGP on dendritic cells, better than other five known types of C-type lectins, the extent of NLGP-Dectin-1 interaction needed further elucidation. The likeliness of the Dectin-1 receptors on dendritic cells to be the potential NLGP-gateway, depended mostly on the sole contribution of Dectin-1 on binding NLGP and the consequent pattern of cytokine secretions, as discussed later. To figure these out, the presence of Dectin-1 receptors was reduced to a specific extent on DC surfaces by post-transcriptional gene silencing (PTGS) and the consequent loss of NLGP-binding as well as shifts in cytokine secretions were measured. Using a 21-mer small interfering RNA (siRNA), single stranded transcripts of Dectin-1 were targeted for Argonaute-mediated cleavage by RNA interference (RNAi) and subsequent destruction to bring down expression levels of Dectin-1 receptor proteins. A non-specific siRNA was used as control. Two groups of mouse BMDCs were transfected with these two siRNAs, whilst keeping another group as non-transfected control. Thus, the extent of RNAi-driven silencing of Dectin-1 protein was checked against two controls, a non-transfected control group and a control siRNA transfected group. Western blotting and densitometry revealed nearly 27.3% and 28.6% of Dectin-1 protein expression after RNAi, compared to the non-transfected/untreated control and the control siRNA transfected groups respectively (Fig. 3a). Hence, the optimized silencing of Dectin-1 through RNAi brought down nearly 72% of its expression. Upon incubating these groups of cells with NLGP-FITC as before, the FITC-fluorescence intensity was found to be significantly low from the Dectin-1 depleted group, compared to those of the two control groups (Fig. 3b). This observation was prominent from MFI values obtained in different experimental replicates, derived from the fluorescence micrographs from both widefield and confocal fluorescence-imaging systems (Fig. 3c, Table S5). Thus, both physical obstructions to Dectin-1-NLGP interactions and decrease in Dectin-1 expression prevent NLGP from binding to dendritic cell surface.

### Binding of NLGP to the Dectin-1 receptors boosts the secretion of IL-12p70 with simultaneous regression of IL-10 from dendritic cells

NLGP promotes aggressive, type 1 immune characteristics for better cancer-combat. It induces a cytokine-milieu to favor inflammation, instigating type 1 immunity. Tendencies of enhancement in IL-12p70 (pro-inflammatory) secretion and reduction in IL-10 (anti-inflammatory) expression to surpass tumor-influences, are NLGP-induced features among dendritic cells. IL-12p35 from *Il12a* gene is the active constituent of IL-12p70 (p35:p40 heterodimer). Thus, IL-12p70 expression follows IL-12p35 production levels. Therefore, varied secretion of two cytokines (IL-10 and IL-12p35) from their usual levels, from mouse BMDCs, were considered as functional readouts to understand the influences of tumor as well as of NLGP on these cells. Roles of Dectin-1 and/or the other C-type lectins under study in mediating physical contact with NLGP were also verified by these DC-functionalities. Tumor-induced conditions were imposed in vitro, using B16 melanoma cell culture supernatant as 1/4th of culture media for these mBMDCs. Influence of this tumor conditioning media (TCM) resulted in upsurged levels of IL-10 alongside abated IL-12p35 production. Further, NLGP supplementation to these tumor-conditioned mBMDCs boosted IL-12p35, with simultaneous repression of IL-10 levels, enhancing type 1 immune features back towards normal (Fig. 2g-i). Levels of these two cytokines were measured in terms of transcripts by reverse transcription, followed by semi-quantitative polymerase chain reaction (RT-PCR) (Fig. 2g), expressed intracellular proteins (flow cytometry) (Fig. 2h) as well as extracellular secretions (enzyme linked immunosorbent assay or ELISA of 48-h mBMDC culture supernatants) (Fig. 2i). To determine the intracellular levels of expression of the secretory cytokines and their variations amongst different cell-groups through flow cytometry, secretion of these proteins were temporarily arrested by ceasing golgi-mediated outward vesicular transport, using Brefeldin A. Following brief Brefeldin A treatment, the cell membranes were permeabilized and the cells were stained with corresponding purified antibodies, followed by the FITC-tagged secondary antibodies and were fixed. Again, considering the whole cell population, the single cells were analyzed for FITC-fluorescence through flow cytometry, as described before. Histograms based on cell-counts were plotted here for each individual group of cells in a linear scale along the Y axis as the percentages of FITC-positive cells have been determined using region statistics along a logarithmic scale on the X-axis (Fig. 2h). Horizontal shifts in the peak-positions of these histograms in the offset-layout indicate variations in cytokine expressions among different groups.

Next, the six aforementioned receptors were neutralized on separate groups of tumor-conditioned mBMDCs, followed by NLGP addition. Deviations in IL-10 and IL-12p35 levels in these groups were compared to a control, where mBMDCs were tumor-conditioned and NLGP treated without any receptor neutralization. Obstacles in NLGP’s access to Dectin-1 brought down transcript-levels of IL-12p35 (Fig. 2j, m) followed by less protein expression (Fig. 2k, m) and sharply reduced extracellular secretion (Fig. 2l, m), whereas all three levels of IL-10 production mounted up (Fig. 2j-m). Any considerable change in production levels of these cytokines, was missing on blocking the other five immunoreceptors (Fig. 2j-m). Collectively, based on the transcription, expression, and secretion profiles of IL-10 and IL-12p35, Dectin-1 was again spotted, neutralization of which could revert the functional aspects of DCs, driven by NLGP, to a significant extent (Fig. 2m).

As Dectin-1 protein expression was depleted again in a group of mBMDCs by RNAi, its effects were studied in NLGP binding to these cells in terms of variations in NLGP-induced cytokine production levels. This time the effects of tumor were studied on DCs, alongside NLGP-driven changes to these tumor conditioned DCs. Also, the extent of NLGP-DC interactions was assessed on depleting Dectin-1 by RNAi in such cells, compared to a non-specific siRNA-transfected control group. Trends of IL-10 and IL-12p35 production by each separate group were taken as readouts as before. As, TCM elevated IL-10 and curtailed IL-12p35 production, addition of NLGP opposed these effects by decreasing IL-10 and enhancing IL-12p35 back towards normal (Fig. 3d-g). However, lowered Dectin-1 expression due to RNAi diminished these activities of NLGP to a large extent, resulting in further upsurge of IL-10 and declined levels of IL-12p35 in the corresponding group (Fig. 3d-g). The control siRNA mostly maintained the effects of NLGP on the tumor-conditioned DCs (Fig. 3d-g). Levels of these cytokines were quantified as transcripts (Fig. 3d, g), cellular proteins (Fig. 3e, g), and secreted proteins (Fig. 3f, g). Thus, diminished expression of Dectin-1 receptors on DC surfaces results in minimization of NLGP’s effects on tumor-influenced DCs, in terms of promoting an inflammatory cytokine milieu (Fig. 3g). Collectively, these observations suggested that the cell-surface interaction between Dectin-1 and NLGP, somehow finds a further way through intracellularly transduced signal(s), to bring about the alterations in these cytokine-secretion levels from DCs.

### β-Glucan chains facilitate the binding of NLGP to Dectin-1 receptor

**Fig. 4:**
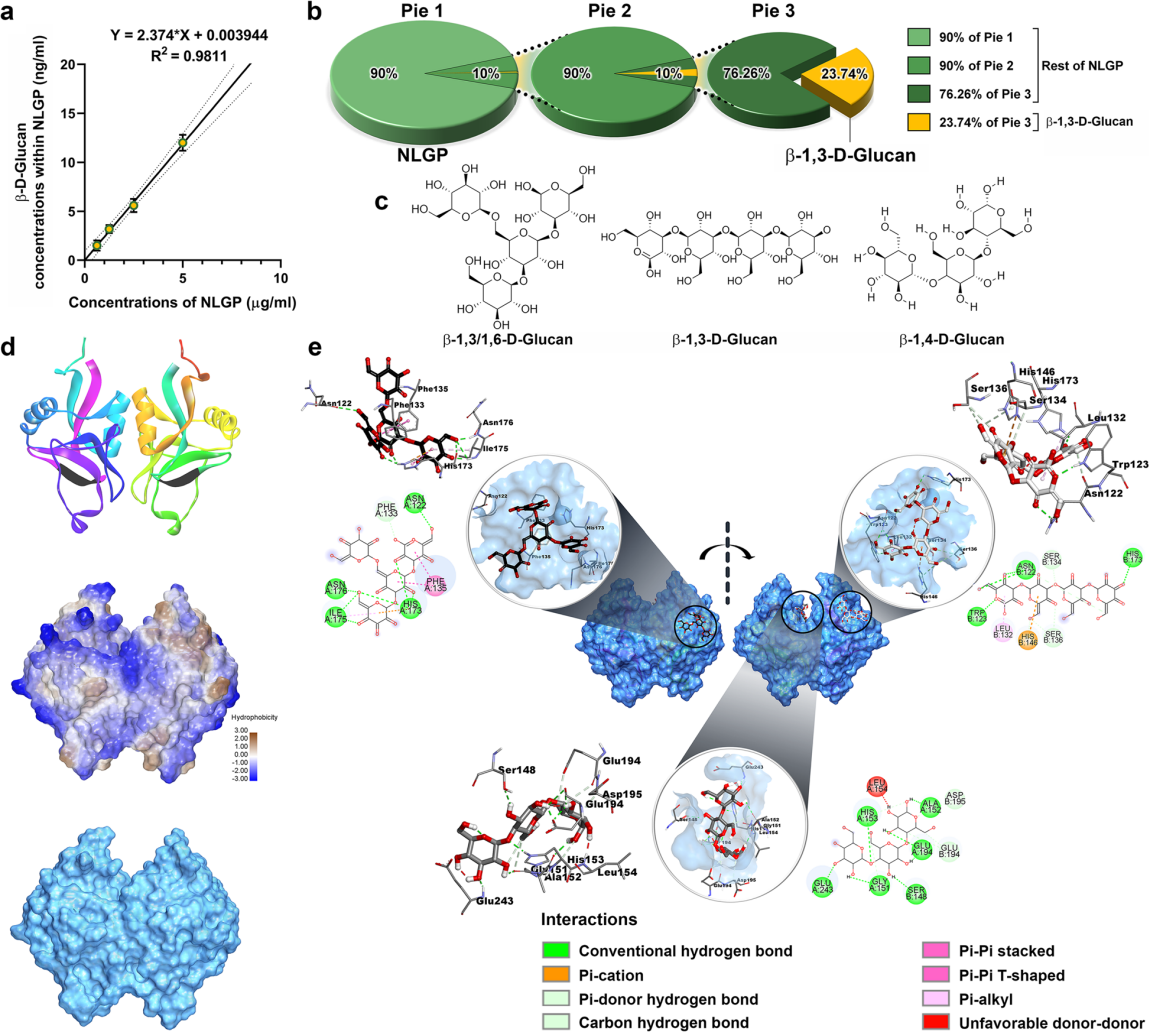
β-D-Glucan structures facilitate the interactions between NLGP and Dectin-1. **a** The regression line and equation between concentrations of NLGP and β-1,3-D-Glucan, determined by ELISA (*n* = 3), as mean ± SD with the R^2^ value. **b** β-1,3-D-Glucan quantified within NLGP in a part of whole pie-chart series, where 10% of the first pie chart (Pie 1) is taken as Pie 2 and 10% of Pie 2 is considered as Pie 3. β-1,3-D-Glucan occupied 23.74% of Pie 3, thereby constituting 0.2374% (w/w) of NLGP. **c** Three abundant short β-D-Glucan structures as flexible small molecules (ligands) for molecular docking. **d** Ribbon (upper), and surface (middle and lower) representations of the dimeric crystal structure of murine Dectin-1 (CLEC7A) (2BPE from RCSB PDB). Cytosolic ITAM sequences highlighted in black, in the ribbon diagram (upper) (ITAM stands for Immunoreceptor tyrosine-based activation motif). Surface model in the middle represents extents of hydrophobicity at different regions. **e** Docked β-D-Glucan structures shown in front and back-views of the Dectin-1 dimer (center) (See also Additional file 4). Localized interactions of Glucans and Dectin-1 amino acids are magnified as 3D (views with the macromolecular surface within circles, and views without the macromolecular surface outside circles) and 2D views. Types of chemical bonds participating in interactions are enlisted in legend at bottom right corner

Adherence of Dectin-1 receptors to their corresponding ligands is facilitated by polymeric chains of Glucose units, called β-D-Glucans/β-Glucans, present on ligand-peripheries. Prominent binding of NLGP to Dectin-1 receptors suggested outward protrusions of such sugar chains (β-Glucans) from this globular glycoprotein. Thus, to find out whether β-D-Glucans are present within the carbohydrate-moiety of NLGP, serially diluted (2 folds) samples of NLGP were immobilized within a microtiter plate and ELISA was performed using the primary antibody against β-1,3-D-Glucan, which confirmed the presence of β-1,3-D-Glucan in each NLGP sample (Fig. 4a). A regression equation was derived between NLGP-concentrations and β-1,3-D-Glucan-content within each sample, from which β-1,3-D-Glucan chains were found to comprise nearly 0.24% of NLGP’s dry weight (Fig. 4b). Structural variants of β-Glucans, other than β-1,3-D-Glucan can be present in NLGP as well.

To understand the molecular details of the interactions within Dectin-1 and NLGP, three abundant short β-Glucan chains (Fig. 4c) were docked onto dimeric murine Dectin-1 molecule (Fig. 4d). The flexible small molecules (three short β-Glucan chains, having 1–3, 1–6; 1–3 and 1–4 linkages respectively) were derived from two longer chains (CHEBI:133,802 and CHEBI:18,246) from ChEBI (Chemical Entities of Biological Interest) of EMBL-EBI (European Molecular Biology Laboratory—European Bioinformatics Institute). The rigid macromolecular structure of mouse Dectin-1 dimer (2BPE) was procured from RCSB PDB (Research Collaboratory for Structural Bioinformatics Protein Data Bank). Based on binding free energies, most stable docking conformations (poses) were screened out of multiple runs for each small β-D-Glucan chain docked in separate pockets of dimeric Dectin-1 (Fig. 4e, Additional file 4). Docked position of the small molecules revealed, participation of Asn122 residues of both Dectin-1 monomers in binding β-1,3/1,6-D-Glucan and β-1,3-D-Glucan chains. As Phe133, Phe135, Ile175, Asn176 etc. from monomer A of Dectin-1 dimer formed H-bonds with β-1,3/1,6-D-Glucan, Leu132, Ser134, Ser136, His173 etc. residues of the monomer B created H-bonds with β-1,3-D-Glucan to stabilise the overall docked structure. Centrally allocated β-1,4-D-Glucan was held by residues from both monomers, including Gly151(A), Leu154(A), Glu194(A), Glu243(A), Ser148(B), Glu194(B), Asp195(B) etc. (Fig. 4e, Table 2, Additional file 4). These interactions uncovered the nature of intermolecular connections, likes of which may occur at the interface of β-D-Glucan extensions of NLGP-surface and extracellular surfaces of Dectin-1 receptors, during NLGP-Dectin-1 association.

**Table 2:** Molecular interactions predicted by docking short β-D-Glucan structures on dimeric mouse Dectin-1

Protein	Dectin-1 (Dimeric) of *Mus musculus* (2BPE from RCSB PDB)					
Small molecule						
	β-_D_-Glc-(1 → 3)-[β-_D_-Glc-(1 → 6)]-β-_D_-Glc-(1 → 3)-β-_D_-Glc		β-_D_-Glc-(1 → 3)-β-_D_-Glc-(1 → 3)-β-_D_-Glc-(1 → 3)-β-_D_-Glc		β-_D_-Glc-(1 → 4)-β-_D_-Glc-(1 → 4)-β-_D_-Glc	
Details	RMSD	91.03	RMSD	93.40	RMSD	43.81
	Binding energy	-9.11 kcal/Mol	Binding energy	-9.16 kcal/Mol	Binding energy	-3.92 kcal/Mol
	Inhibition constant (Ki)	211.70 nM	Inhibition constant (Ki)	192.34 nM	Inhibition constant (Ki)	1.34 mM
H-bonds formed	6		7		11	
Interacting amino acids of Dectin-1	Asn122(A), Phe133(A), Phe135(A), His173(A), Ile175(A) & Asn176(A)		Asn122(B), Trp123(B), Leu132(B), Ser134(B), Ser136(B), His146(B) & His173(B)		Gly151(A), Ala152(A), His153(A), Leu154(A), Glu194(A), Glu243(A), Ser148(B), Glu194(B) & Asp195(B)	
	(Where A and B are the monomeric subunits of dimeric Dectin-1)					

*Abbreviations: RCSB* Research Collaboratory for Structural Bioinformatics, *PDB* Protein Data Bank, *Glc* Glucan, *RMSD* Root Mean Square Deviation

### NLGP-Dectin-1 interaction initiates an intracellular signal to NFκB through PKCδ, CARD9, Bcl10 and MALT1-signaling axis with simultaneous internalization of NLGP

**Fig. 5:**
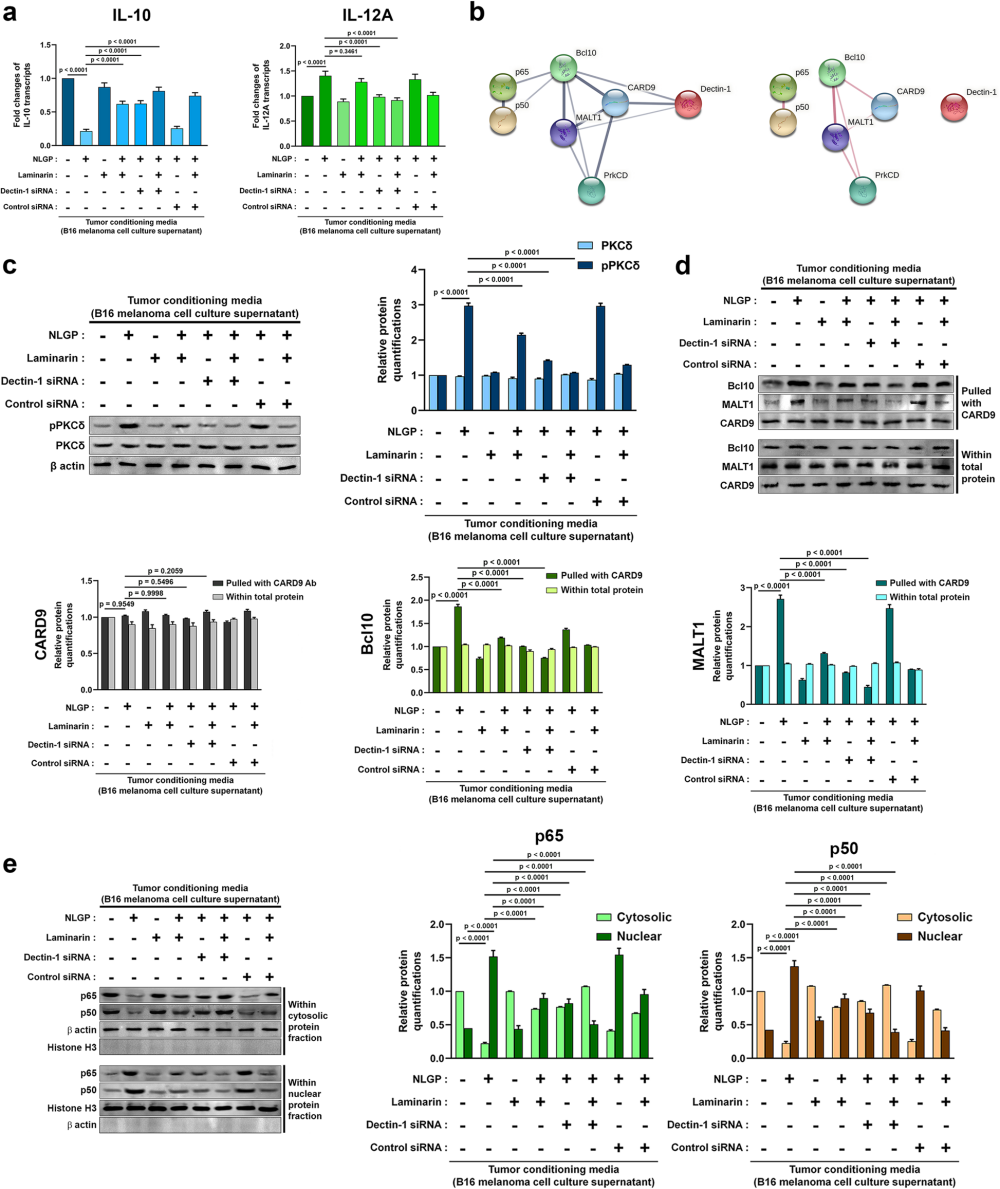
NLGP-Dectin-1 interaction transduces a PKCδ-mediated intracellular signal to NFκB subunits through CARD9-Bcl10-MALT1-based signaling axis. **a** Quantitative PCRs measuring IL-10 and IL-12A or IL-12p35 transcripts on NLGP treatment to tumor-conditioned mBMDCs and effects of inhibiting NLGP-Dectin-1 interactions subsequently by Laminarin, RNAi-based Dectin-1 depletion and both. β actin was taken as endogenous control, fold changes were deduced by Livak method (2.^−ΔΔCt^) from replicates (*n* = 3) (data are represented as mean ± SD). **b** General interactions among probable second messengers of NLGP-Dectin-1 association-induced signals in DCs, deduced from STRING (Search Tool for the Retrieval of Interacting Genes/Proteins) (Ver. 11) based on their reported physical (physical network) (right) and distal (full network) (left) associations. The PrkCD node stands for Protein kinase C δ (PKCδ). **c** Western blotting to measure PKCδ and phospho-PKCδ (pPKCδ) in same experimental mBMDC groups as (**a**). β actin was kept as loading control (*n* = 3) (data from replicates are represented as mean ± SD). **d** Western blots to quantify levels of Bcl10 and MALT1 (both bound to CARD9) in these groups by co-immunoprecipitation, as CARD9 was pulled with its antibody out of total cell protein lysates. Levels of CARD9 and all three proteins (within total protein lysates) were kept as positive control (upper left). Data from replicates (*n* = 3) are represented as mean ± SD (upper right, lower left and lower right). **e** Western blots, measuring p65 (also known as RelA from the *Rela* gene) and p50 (of NFKB1, transcribed from the *Nfkb1* gene) proteins within cytosol and nuclei in these mBMDC groups. β actin and Histone H3 were taken as loading controls for cytosolic and nuclear proteins respectively. Data from replicates (*n* = 3) are represented as mean ± SD (middle and right). Numerical data of (**a**) were tested by one-way ANOVA (*F* = 126.2 and 29.19, df = 23 and 23) followed by Tukey’s multiple comparisons test, and those of Figures (**c**), (**d**) and (**e**) were tested by two-way ANOVA followed by Tukey’s multiple comparisons test. Normal distribution of the data were verified before each test (See also Fig. S2e-l)

After knowing that NLGP interacts with Dectin-1 receptors on mouse dendritic cell-surface, the study moved to delineate the consequent intracellular events. Taking tumor-conditioned mBMDCs as control, effects of NLGP was tested (group 2) (Fig. 5). Obstacles towards Dectin-1 were gradually increased through antagonist Laminarin, RNAi based Dectin-1 depletion and both (groups 4–6) (Fig. 5). Control groups for the inhibitors included Laminarin addition without NLGP, and control siRNA-transfection with and without adding Laminarin. Quantitative PCR revealed nearly 4.5 fold-decrease of IL-10 and 1.5 fold-increase of IL-12p35 transcripts due to NLGP addition to tumor-conditioned cells, both of which were gradually counterbalanced by Laminarin, Dectin-1 siRNA and their combined inhibitory effects (Fig. 5a). Signals initiated by Dectin-1 receptors primarily include PKCδ, CARD9, Bcl10, MALT1, NFκB, and their associated interacting protein partners as second messengers and transcription factors to alter cytokine levels. General relations amongst these molecules were elucidated using the Search Tool for the Retrieval of Interacting Genes/Proteins (STRING) database based on their reported physical and indirect associations (Fig. 5b). NLGP escalated PKCδ phosphorylation in tumor-conditioned mBMDCs, which diminished successively on increasing obstacles to cell-surface Dectin-1 by Laminarin, Dectin-1 siRNA, and both. Whereas expression of PKCδ, followed similar levels throughout these conditions (Fig. 5c). Physical association of CARD9, Bcl10, and MALT1 was tested by co-immunoprecipitation. CARD9 was equally immunoprecipitated from total cell protein lysates of each group, but Bcl10 and MALT1 attached to CARD9 increased nearly 2 and 3 folds respectively on NLGP addition to the tumor-conditioned cells, suggesting the trimolecular complex formation because of the NLGP-transduced signal (Fig. 5d). Subsequent rise of NFκB monomers i.e., p65 (also known as RelA from the *Rela* gene) and p50 (of NFKB1, transcribed from the *Nfkb1* gene) was also seen following NLGP treatment among nuclear proteins, alongside their decrease within cytosol (Fig. 5e). Obstacles in NLGP-Dectin-1 interactions caused by the inhibitory agents steadily obstructed this nuclear translocation of NFκB and the preceding event of trimolecular complex formation (Fig. 5d, e).

**Fig. 6:**
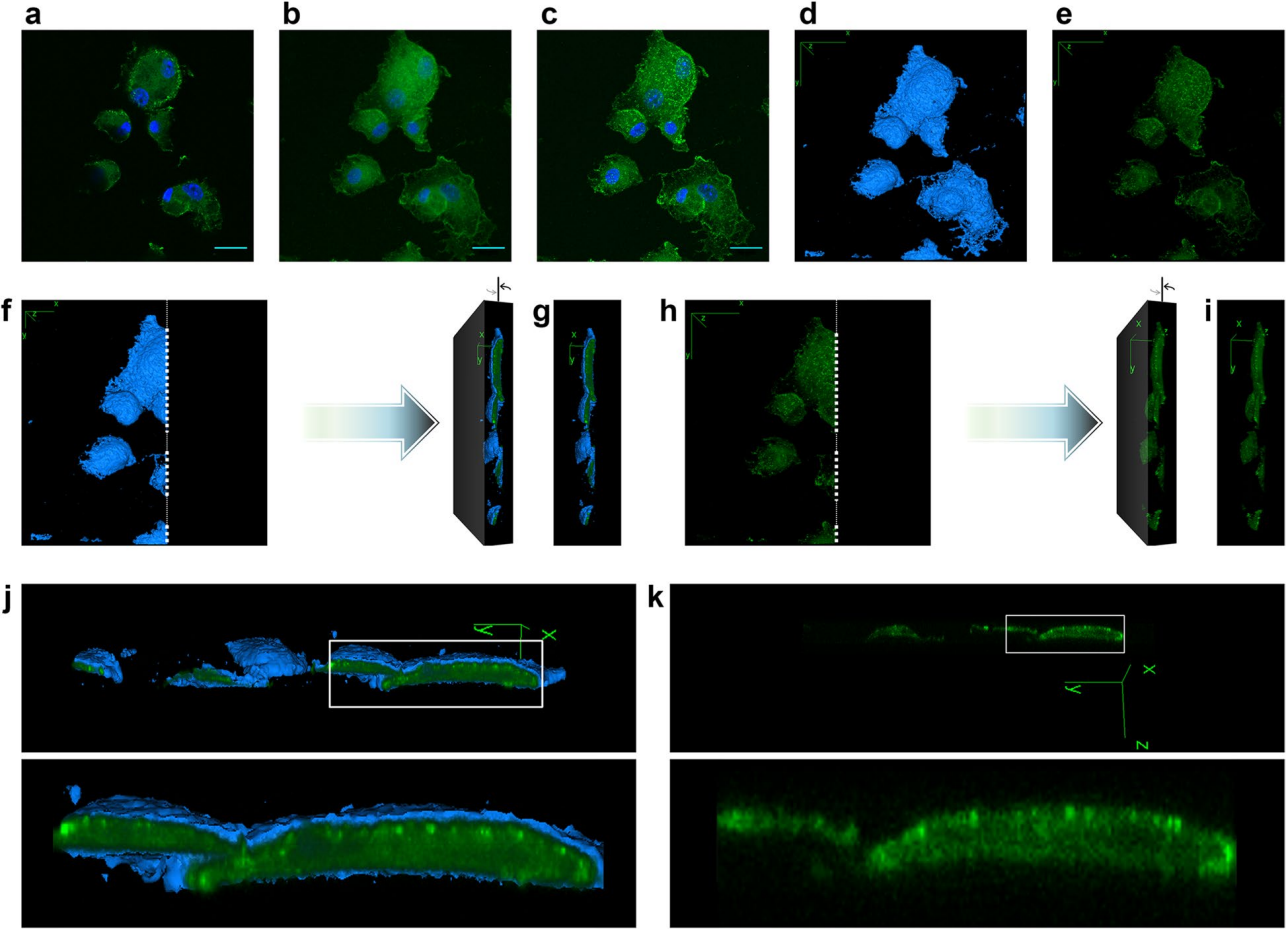
Presence of NLGP within the cytosol alongside its accumulation by the membrane of mouse dendritic cells, reveals its intracellularization following the interaction(s) with Dectin-1 receptors. **a** A 2D fluorescence-micrograph of mBMDCs incubated with NLGP-FITC for 1 h, captured through confocal microscope (nuclei stained with DAPI). **b** Sum slices type 2D Z-projection of the field, derived by flattening the Z-stack (individual images of successive layers of the Z-stack are shown in Additional file 5, sequentially from above along a specific thickness interval) through sum slices method. **c** Maximum intensity type 2D Z-projection of the field (scale bars:10 µm in (**a**), (**b**) and (**c**)). **d** Surface view of the field (cells’ exterior surfaces shown in blue/cyan) (See also Additional file 6). **e** FITC channel-based volume view of the field (See also Additional file 7). **f** Longitudinal section was obtained through the composite view (surface view + volume view) of the cells, along white dots. Left side of this section was rotated clockwise around the Y-axis or the vertical axis revealing cell-interiors along section. **g** Rotated composite view, cut parallel to Y–Z plane. **h** Longitudinal section through volume view of the field, rotated to reveal cell interiors. **i** Rotated volume view cut parallel to Y–Z plane (See also Additional file 8). **j** Rotated composite view of the field (as in (**g**)), positioned horizontally (upper) and an area along the section, revealing the cell interiors is marked. The marked zone is magnified further (lower) revealing NLGP-FITC within the cells. **k** Slice (orthoslice) obtained by rotating the volume view, cut parallel to Y–Z plane (as in (**i**)), placed horizontally (upper), revealing intracellularized NLGP-FITC, within the magnified marked region (lower)

Meanwhile, NLGP enters the cytosol of dendritic cells, which was observed through a confocal microscope. Mouse BMDCs were incubated with NLGP-FITC for 1 h, washed and fixed and the FITC-emission was captured in confocal fluorescence-micrographs (Fig. 6a-c). 2D images captured along consecutive layers along a specific thickness-interval (through Z-axis) of the same field were stacked as a 3D representation (Z-stack) (Additional file 5). This Z-stack was flattened along Z-axis in different ways producing sum slices (Fig. 6b) and maximum intensity (Fig. 6c) based 2D representations (Z-projections) of the field. Extracellular surfaces were seen in the surface view (Fig. 6d, Additional file 6) of the cells from the Z-stack and fluorescent-signal emitting points were collectively observed in their volume view (Fig. 6e, Additional file 7). Superimposition of both surface and volume views of these cells were taken as a composite view. A longitudinal section was obtained, parallel to the Y–Z plane in both the composite (Fig. 6f, g) and volume views (Fig. 6h, i, Additional file 8) of the field to cut open the cells’ interior cytosol, which were revealed by rotating the cut-sections clockwise around the Y-axis or the vertical axis (Fig. 6f-I, Additional file 8). A prominent zone revealing the cells’ interiors was marked within these rotated views of the section and was magnified (Fig. 6j, k). Accumulation of fluorescent-signals along cell surface displayed NLGP’s binding to cell surface Dectin-1 receptors (Fig. 6a). Whereas, prominent signal captured from the cytoplasm (Fig. 6j, k) unveiled NLGP’s entry within the cells, possibly via Dectin-1 receptor mediated endocytosis, which might have a role in attenuating the aforementioned intracellular signal.

### NLGP driven Dectin-1 induced signal causes contrasting regulation in *Il10* and *Il12a* gene transcriptions through different dimeric combinations of NFκB

Next, possibilities of NFκB-based transcriptional regulation of *Il10* and *Il12a* genes due to NLGP-Dectin-1 transduced signal were explored within nuclei of tumor-conditioned mBMDCs, following nuclear translocations of the NFκB subunits, p65 and p50. Accordingly binding of p65 and p50 were checked on corresponding transcription factor binding motifs (TFBMs) or operator sequences of *Il10* and *Il12a* genes by chromatin immunoprecipitation amongst the same mBMDC groups as described previously. The p65 and p50-bound chromatins were isolated from each group and *Il10* and *Il12a* gene TFBM-specific primers were used to amplify them from these chromatins by PCR, for detection and quantification, as described in Methods.

**Fig. 7:**
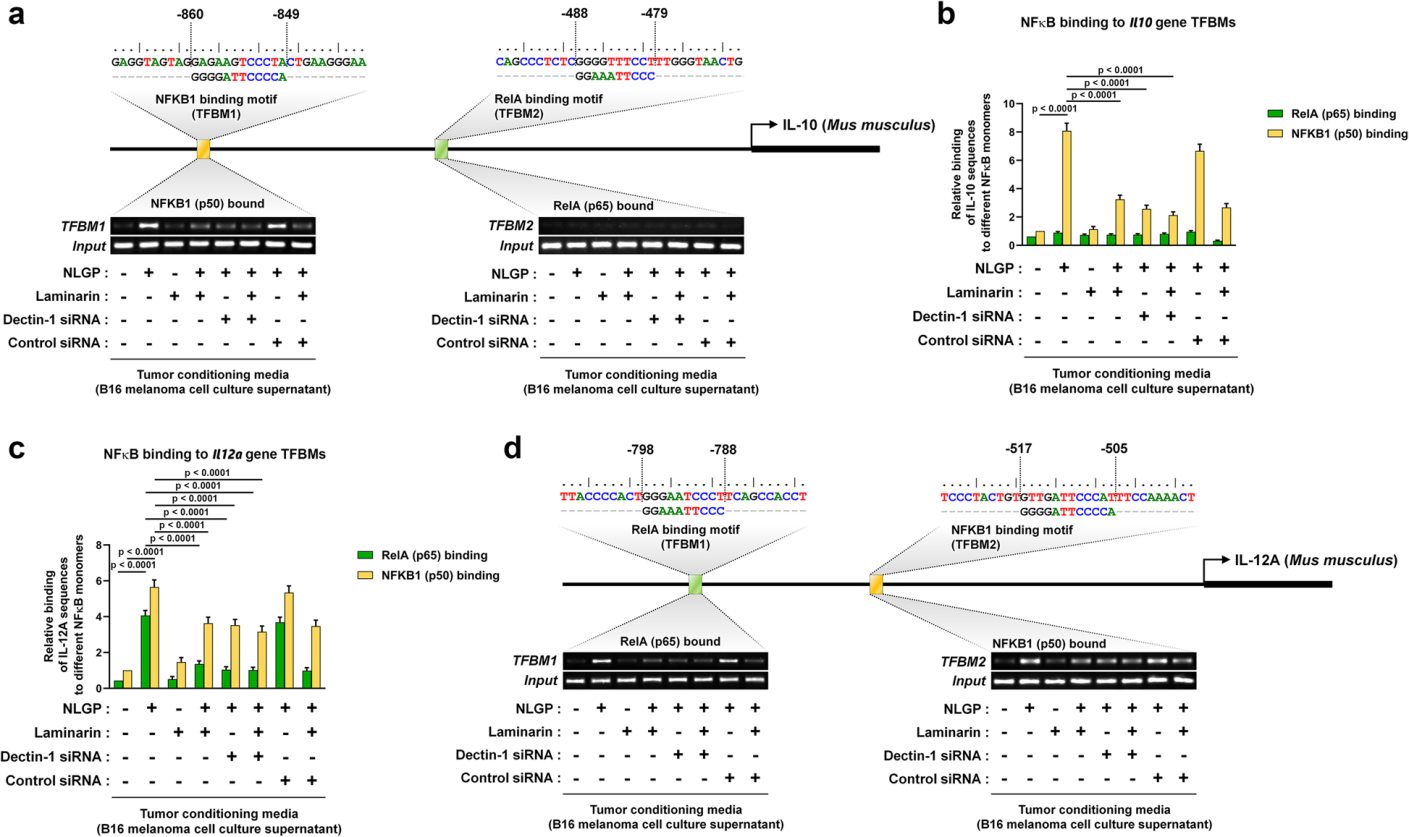
Selective binding of NFκB monomers to the genes of *Il10* and *Il12a* were explored by Chromatin Immunoprecipitation studies.** a** Pictorial representation of p50 and p65 binding to their corresponding TFBMs on mouse *Il10* gene, due to NLGP transduced signal, followed by obstructions towards Dectin-1. TFBM sequences identified by sequence alignment are shown (distance mentioned from the transcription start site or TSS) (above) along with agarose gel images of relative amplifications of protein-bound TFBM (below) (TFBM stands for transcription factor binding motif). **b** Data from different replicates (*n* = 3) are represented as mean ± SD. **c** Data for comparative binding of p65 and p50 to *Il12a* gene TFBMs from replicates (*n* = 3), as mean ± SD. **d** Binding of p65 and p50 to *Il12a* TFBMs, represented pictorially in the same way as for *Il10* in (**a**). Numerical data of (**b**) and (**c**) were tested by two-way ANOVA, followed by Tukey’s multiple comparisons test. Normal distribution of the data were verified before each test (See also Fig. S3)

NLGP induced significant p65-binding to the *Il12a* gene-TFBM in tumor-conditioned mBMDCs, which decreased gradually on hampering Dectin-1-NLGP interactions by Laminarin, Dectin-1 downregulating siRNA and both (Fig. 7c, d), whereas a non-significant basal level of p65-binding to *Il10*-TFBM was found in each varying circumstance (Fig. 7a, b). However, NLGP-driven binding of p50 was prominent to both *Il10* and *Il12a* TFBMs, which again reduced gradually with increasing obstructions in NLGP-Dectin-1 interactions (Fig. 7a-d). So, the signal transduced from NLGP-Dectin-1-association favors p65 binding only to the *Il12a* TFBM whereas, on being driven by the same signal, p50 binds prominently with both the *Il10* and *Il12a* TFBMs. The p65 and p50 subunits of NFκB exist in dimeric conformations. Thus, due to this signal, p50 binds *Il10*-TFBM as a homodimer, whereas the p65:p50 heterodimer binds to the *Il12a* TFBM. Absence of the transcription activation domain (TAD) in p50 renders the p50:p50 homodimer the canonical repressor, which might have a major role in suppressing *Il10* gene’s transcription and consequent decreased IL-10 expression. Whereas, due to presence of TAD in p65 the p65:p50 heterodimer probably upregulates *Il12a* gene’s transcription with subsequent elevations in IL-12p35 and IL-12p70 expression.

**Fig. 8:**
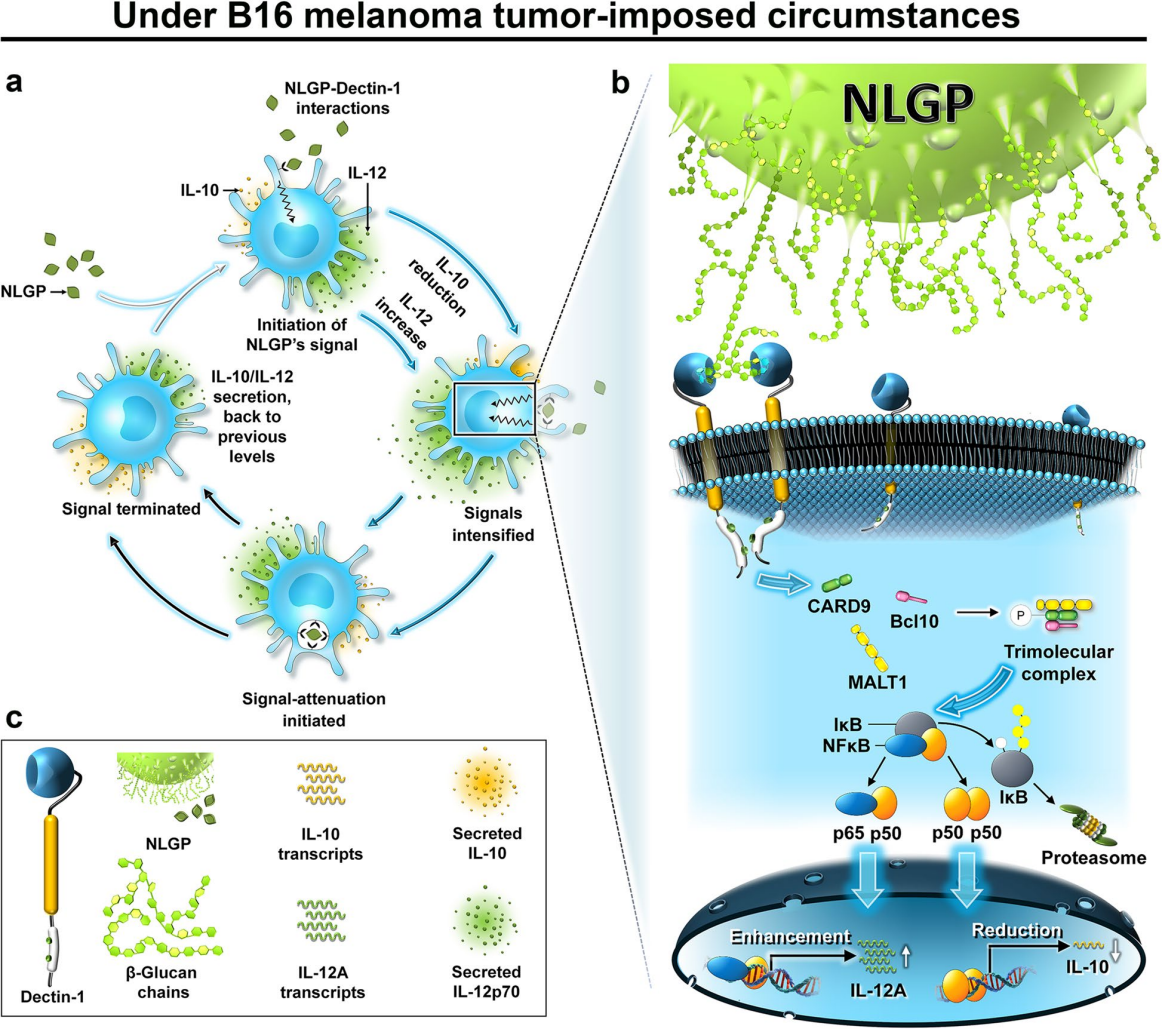
Pictorial summary from the findings. **a** Under the influences of B16 melanoma, murine dendritic cells secrete IL-10 (anti-inflammatory) and IL-12 (IL-12p70, mainly depending on its constituent IL-12p35 from *Il12a* gene) (pro-inflammatory) at their corresponding tumor-imposed levels. Neem Leaf Glycoprotein (NLGP) binds to Dectin-1 receptors, on these cells and initiates an intracellular signal transduction cascade, which reduces IL-10 secretion and simultaneously increases secretion level of IL-12p70 from these cells. As this signal intensifies gradually, reduction of IL-10 and enhancement of IL-12p35 becomes prominent at their secretory levels, thereby initiating type 1 immune-commitment. However, intracellularization of NLGP might play a role in attenuation of this signal later on, which eventually would terminate this signal. **b** As the signal initiated by NLGP-Dectin-1 receptor association within these dendritic cells and its intracellular transduction was studied, it was found that upon proximity to these cells, NLGP binds Dectin-1 receptors through its protruded β-D-Glucan chains. Consequently, a signal is delivered to CARD9, through phosphorylation of PKCδ (not shown in this representation). CARD9, on being phosphorylated attaches itself to Bcl10 and MALT1, forming the trimolecular CARD9-Bcl10-MALT1 (CBM) complex. This complex activates NFκB in the canonical way. Thus, upon phosphorylation, ubiquitination and subsequent proteasomal degradation of the corresponding IκB components, the canonical NFκB dimers, p65:p50 and p50:p50 translocate into the nuclei of these cells. Within the nuclei the enhancer p65:p50 dimer raises *Il12a* gene’s transcription, whereas the repressor dimer p50:p50 decreases the transcription rate of the *Il10* gene. Following translation, modifications, secretion etc., such regulated levels of these cytokines are reflected as an uprise in secretion of the pro-inflammatory cytokine IL-12p70, along with depleted secretion of the anti-inflammatory IL-10 secretion from these dendritic cells. **c** Diagrammatic legend of the visual perspectives in (**a**) and (**b**)

Thus collectively, even under the detrimental influences of tumor microenvironment, NLGP initiates an inflammation-favoring anticancer type 1 immune-commitment through upregulation of pro-inflammatory IL-12p70 and reduction of anti-inflammatory IL-10 secretion from murine dendritic cells. Through its β-D-Glucan chains, NLGP binds to the Dectin-1 receptors on these dendritic cells and initiates an intracellular signal, which propagates by phosphorylation of PKCδ, followed by a trimolecular complex formation of CARD9, Bcl10 and MALT1. As this complex activates the canonical NFκB-driven transcriptional regulation, the p65:p50 enhancer dimer of NFκB upregulates *Il12a* transcription and the p50:p50 repressor dimer downregulates *Il10* transcription within the nuclei. Internalization of NLGP within the dendritic cells takes place, which might happen due to the endocytic properties of Dectin-1 receptors and might also lead to the signal’s attenuation (Fig. 8). However, no link between intracellularization of NLGP and the Dectin-1 receptors’ endocytic properties were addressed in this study, nor this internalization was investigated to be a specific reason for attenuation of the NLGP-induced signal.

## Discussion

The astronomical heterogeneity of the adaptive immunoreceptor-paratopes (T cell receptors or TCRs and Immunoglobulins or BCRs) confers near-absolute precision in immunogen-detection and such diverse paratopes are being sequenced till date by the ‘Adaptive Immune Receptor Repertoire Community (AIRRC)’ [30]. However, the phylogenetically ancient ‘innate immunoreceptors’ present on the APCs, which act as the first-line sensors for threat detection recognize some conserved molecular patterns present on exogenous threats, named ‘pathogen associated molecular patterns’ (PAMPs) or ‘microbe associated molecular patterns’ (MAMPs) as immunogenic signatures [31]. The ‘danger theory’ by Polly Matzinger explained, rather than being evolutionarily conserved, the threatening molecular patterns from both self and foreign sources depend on their damage-inflicting capability, which were thus renamed as ‘damage associated molecular patterns’ (DAMPs) [32]. The innate immunoreceptors also called as ‘pattern recognition receptors’ (PRRs) [31] include membrane-embedded molecules like Toll like receptors (TLRs), C type lectin receptors (CLRs), cyclic GMP-AMP synthase (cGAS) etc. as well as intracellular categories like NOD-like receptors (NLRs), RIG-I-like receptors (RLRs), AIM2-like receptors (ALRs) etc. [33].

With continuous acquisition of the vast literature on NLGP’s immunomodulatory functions in relation to cancer [10–22, 24, 25, 27, 28, 34], we needed to identify the immunoreceptor that initiates NLGP’s action. The plant glycoprotein NLGP does not pose a threat to the host immunity due to its insignificant cytotoxicity [12]. As this ligand should lack PAMPs/DAMPs, the corresponding immunoreceptor was searched based on its binding-affinity towards glycoprotein ligands. The sugar-domains (glycans) associated to individual amino acids of glycoproteins usually bind the carbohydrate-binding proteins (lectins). Among 12 or more categories of lectins studied till date, the C-type lectins act as Ca^++^ dependent carbohydrate binding receptors on APC surfaces, being the second most prominent category of PRRs after TLRs [34]. Several functions of these receptors mould the tumor-immune environment in important ways as well [35–38]. Thus, six well known C-type lectin receptors (CLRs or CLECs) were screened for NLGP-binding. As binding of NLGP on dendritic cell-surfaces was prominently visualized by the fluorescent FITC-tag (attached to it later), the extent of the attached NLGP visibly diminished to a great extent on pre-occupying the Dectin-1 receptors on DC surfaces. NLGP’s adherence to the DCs remained nearly unchanged on pre-occupying the other five receptors similarly. As the flow cytometric analyses alongside knockdown assays of Dectin-1 by RNAi also yielded outcomes demonstrating NLGP-Dectin-1 attachment on DC surfaces, Dectin-1 was considered to be the putative candidate receptor.

Polysachharides with dextrorotatory β-Glucose units, named β-D-Glucans are indispensable for ligand-binding to Dectin-1 [39]. Found on the cell walls of fungi, yeast, etc. as well as in cereals and plants, β-D-Glucan chains (having mostly β-1 → 3 glycosidic linkages and β-1 → 6 branchpoints) generally exist as peripheral hairlike protrusions from the ligand-surfaces. Accordingly, the β-1,3-D-Glucans in NLGP (nearly 0.24%) speculatively form a woolly sugarcoat around the globular glycoprotein surface, creating the Velcro-like interface of adhesion between NLGP and dendritic cells through Dectin-1.

However, in nature these β-D-Glucan structures can exist as both branched an unbranched sugar chains, having different combinations of 1 → 4, 1 → 3, 1 → 6 and rarely 1 → 2 glycosidic linkages as well. Triple helical turns and the resulting topographies observed in β-1,3-D-Glucans, would give rise to epitopes for binding specific antibodies. Due to their high affinities towards Dectin-1, presence of these β-1,3-D-Glucans were checked by the Anti-1,3 beta Glucan antibody [2G8] (RRID:AB_2923478), from Abcam (Cat# ab233743) and confirmed. But, some of the structural variants of β-D-Glucans, without 1 → 3 β-glycosidic bonds may also be present in the hypothetical coat of these sugars on NLGP’s surface. Functionalities of such sugar chains may span beyond Dectin-1 binding, as they can participate in binding other molecules, as well as alter the overall hydrophilicity of NLGP.

Dectin-1 receptors have a ligand-binding extracellular ‘C-type lectin-like domain/ carbohydrate recognition domain’ (CRD) and an intracellular ‘immunoreceptor tyrosine-based activation motif’ (ITAM). Unlike most immunoreceptors, Dectin-1 has a single ITAM bearing the sequence ‘…YXX[L/I]X_6-9_YXXX[L/I]…’ (X represents any amino acid) [40].

In collaboration with the TLR2:TLR6 dimer, Dectin-1 is known to engulf fungal antigens, inducing a Raf-1 mediated signal that promotes NFκB [41]. As the preliminary observations nullified the participation of TLRs in NLGP-signaling, we focused on the canonical signaling pathway initiated by Dectin-1, independent of TLR-assistance. Here, upon binding its ligand, the membrane proximal tyrosine residue of the Dectin-1 ITAM gets phosphorylated by Src kinase, leading to local accumulation of Dectin-1 molecules along the plasma membrane, near the ligand [42]. Syk then docks at the site created by two phospho-tyrosines from two adjacent Dectin-1 molecules [40] and creates a multiprotein complex with SLP-65 (also known as BLNK) and Tec kinases [43]. Consequently, PLCγ recruited by SLP-65 is phosphorylated by Tec kinases [43], which in turn hydrolyzes PIP_2_, to generate IP_3_ and DAG [43]. DAG in turn phosphorylates PKCδ and sends it into the cytosol. Within the cytosol pPKCδ activates CARD9 by phosphorylation at Thr231 [44], which then attaches itself to Bcl10 through the caspase activation and recruitment (CARD) domain by CARD-CARD interaction. Bcl10 also adheres to the paracaspase MALT1, forming the CARD9-Bcl10-MALT1 complex (CBM) due to NLGP-induced Dectin-1-downstream signals. Following oligomerization of the CBM complex, Bcl10 activates NEMO (also known as IKKγ) either by phosphorylation through TRAF6 and TRAF2 as adaptors and RIP2 as associate kinase [45, 46], or by ubiquitination at Lys63 of NEMO by Ubc13 and/or Uev1A [47]. Activated NEMO exposes the activation loop serines of the catalytic IκB kinases (IKKs) (Ser177 and Ser181 on IKKβ and Ser 176 and Ser180 on IKKα) [48, 49], which are then phosphorylated by transautophosphorylation or by TAK1 and/or NIK [45]. Further phosphorylations at Ser740 in IKKβ and Ser68 in NEMO, dissociates NEMO from the IKK complex which gets re-associated by Cdc37 and HSP90 chaperones and phosphatases [45]. Roughly a 33 amino acid consensus sequence, termed as ‘ankyrin repeat’ can be found in multiple copies of the ‘traditional’ and ‘precursor’ IκB molecules, which enable them to mask the nuclear localization signals (NLSs) of the NFκB dimers, sequestering them inactive in cytosolic NFκBsomes [45, 50]. As the traditional IκBs include IκBα, IκBβ and IκBε, precursor IκBs exist as the C-terminal sequence of some NFκB polypeptides to be cleaved and digested by 20S proteasome on NFκB activation [45]. Phosphorylation of the IκB molecules by the IKK and subsequent proteasomal degradation of IκB molecules, free the NFκB dimers to enter the nucleus and bind gene enhancers. Out of the twelve dimeric combinations observed in vivo from the five known NFκB monomers, the p65:p50 heterodimer appears to be the most stable and frequent [50]. In the canonical NFκB-based transcription-regulation, this heterodimer acts as an activator, whereas the p50 monomer lacking the ‘transcription activation domain’ (TAD), renders the p50:p50 homodimer a repressor [50]. NLGP induces translocation of the p65:p50 and p50:p50 dimers into the nuclei. IKKβ-driven phosphorylation of Ser923 and Ser927 near the C-terminus of p105 (cleaved off at amino acid 420) generates p50 from its precursor, whereas mostly IKKβ and also IKKα phosphorylate the Ser32 and Ser36 of IκBα, to release the p65:p50 dimer [45]. Due to NLGP-Dectin-1 signaling, the p65:p50 heterodimer enhances transcription of *Il12a* gene, with simultaneous restriction on *Il10* gene transcription by p50:p50.

Constrained *Il10* transcription limits the risk of instigation of the host immune system towards tumor-promoting type 2 phenotype. *Il12a* transcribes and translates to IL-12p35, which on getting upregulated binds IL-12p40 producing substantially more amount of the pro-inflammatory cytokine IL-12p70. Elevated IL-12p70 secretion brings about inflammation promoting changes amongst the immune-components including higher levels of IFNγ production and augmented T cell cytotoxicity inducing type 1 immune-commitment. Thus, by regulating these two cytokines in DCs, NLGP orchestrates far-leading consequences by charging the whole aggressive immune-arsenal against the tumor, leading to tumor-growth restriction in vivo.

Also, intracellular signals of NLGP are known to prevent STAT3 phosphorylation in immune cells, thereby rescuing macrophages from M2 phenotypes [15] and reducing the immunosuppression by myeloid derived suppressor cells (MDSCs) [34] in previous reports. Phosphorylations of Tyr705 and Ser727 of STAT3 by JAK1 and MAPKs respectively, cause pSTAT3 dimerization by phosphotyrosine-SH2 domain interactions [51]. Prevention of STAT3 phosphorylation possibly by SOCS3 manifests the NLGP-induced signals within the host, also leading to type 1 immunity. Crosstalk between multiple signals induced by NLGP especially in the myeloid cells opens up a new domain for study.

The extensive sphere of glycosylation spreads beyond receptor-ligand interactions including the aspects of immune cell homeostasis and migration [52]. This study aims to bridge the understandings of NLGP-induced regulations of cell homeostasis leading to the control of type-1/type-2 balance with the initial signaling gateway of NLGP in context to protein glycosylation. The important aspect of glycan-contained ‘information’ in the management of carcinogenesis will further enrich the field of cancer glycoimmunology.

## Conclusion

In summary, in search of the initial cellular and molecular interactions that disseminate as systemic anticancer immunomodulation of Neem Leaf Glycoprotein, we found Dectin-1 as the putative receptors on dendritic cells. Further, we came to know that, as NLGP binds these Dectin-1 receptors on the surfaces of the dendritic cells via protrusive chains of β-Glucans on its surface, an intracellular signal is initiated within these dendritic cells. This signal phosphorylates PKCδ within the cytosol and is transduced further through the formation of a trimolecular complex of CARD9, Bcl0 and MALT1. Later, this signal activates the canonical pathways of NFκB-based regulations of cytokine gene-transcriptions. As the NFκB dimers (p65:p50 and p50:50) translocate into DC nuclei, the heterodimeric activator dimer p65:p50 binds and enhances *Il12a* gene transcription, resulting in upregulation of IL-12p35 and thus the pro-inflammatory cytokine IL-12p70. Simultaneously, as the repressor homodimer p50:p50 represses *Il10* transcription, the anti-inflammatory cytokine IL-10 is downregulated. Thus, the systemic immune environment takes a turn towards the pro-inflammatory type 1 immunity, which intensifies further through the responding immune cells. This intense type 1 immunopolarization alongside the increase in cytotoxicity of activated CD8^+^ T cells combine to provide the necessary and robust anti-tumor boost to the otherwise suppressed host-immunity through NLGP, regressing cancerous tumors and metastasis.

Substantially, these would help to explain the observed physiological potential of natural products in upcoming future. This study may pioneer in connecting these potent features to their underlying basics amongst the known interactome of biomolecules. Consequently, the causative events would be identified within cells and tissues.

### Supplementary Information

**Supplementary Material 1.**

**Supplementary Material 2.**

**Supplementary Material 3.**

**Supplementary Material 4.**

**Supplementary Material 5.**

**Supplementary Material 6.**

**Supplementary Material 7.**

**Supplementary Material 8.**

**Supplementary Material 9.**

## Data Availability

No datasets were generated or analysed during the current study.

## Abbreviations

AIRRC: Adaptive immune receptor repertoire community
ALC: Average local confidence
ALR: Absent in melanoma 2 (AIM2)-like receptor
ANOVA: Analysis of variance
APC: Antigen presenting cell
Bcl10: B cell lymphoma/leukemia 10
BCR: B cell receptor
BLNK: B cell linker
BMDC: Bone marrow derived dendritic cell
BSA: Bovine serum albumin
C12: Carbon isotope with an atomic mass of 12
C18: Silica containing 18 carbon atoms
C57BL/6J: C57 black 6 inbred parental substrain of mice from Jackson Laboratory
CARD: Caspase activation and recruitment domain
CARD9: Caspase activation and recruitment domain-containing protein 9
CBM: CARD9-Bcl10-MALT1
CCR7: C-C chemokine receptor type 7
CD8: Cluster of differentiation 8
CD16: Cluster of differentiation 16
CD28: Cluster of differentiation 28
CD32: Cluster of differentiation 32
CD62L: Cluster of differentiation 62L
CD69: Cluster of differentiation 69
CD80: Cluster of differentiation 80
CD83: Cluster of differentiation 83
CD86: Cluster of differentiation 86
CD107a: Cluster of differentiation 107a
CD163: Cluster of differentiation 163
CD205: Cluster of differentiation 205
CD206: Cluster of differentiation 206
CD209: Cluster of differentiation 209
CD369: Cluster of differentiation 369
CD370: Cluster of differentiation 370
Cdc37: Cell division cycle 37, HSP90 cochaperone
cGAS: Cyclic GMP-AMP synthase
ChEBI: Chemical entities of biological interest
ChIP: Chromatin-immunoprecipitation
CLEC: C-type lectin domain
CLEC4L: C-type lectin domain family 4, member L
CLEC6A: C-type lectin domain family 6, member A
CLEC7A: C-type lectin domain family 7, member A
CLEC9A: C-type lectin domain family 9, member A
CLR: C-type lectin receptor
CRD: Carbohydrate recognition domain
C_t_: Cycle-threshold
CTCF: Corrected total cell fluorescence
CTLA4: Cytotoxic T lymphocyte-associated protein 4
DAG: Diacylglycerol
DAMP: Damage associated molecular pattern
DAPI: 4′,6-Diamidino-2-phenylindole
DC: Dendritic cell
DC-SIGN: Dendritic cell specific intercellular adhesion molecule-3-grabbing non-integrin
DIA: Data independent acquisition
DMEM: Dulbecco's modified eagle medium
DMSO: Dimethyl sulfoxide
EDTA: Ethylenediaminetetraacetic acid
ELISA: Enzyme linked immunosorbent assay
EMBL-EBI: European molecular biology laboratory-European bioinformatics institute
EPD: Eukaryotic promoter database
ESI: Electrospray-based ionization
FBS: Fetal bovine serum
FITC: Fluorescin isothiocyanate
FWHM: Full width at half maximum
GATA3: GATA binding protein 3
GM-CSF: Granulocyte macrophage-colony stimulating factor
HCD: High energy collision-induced dissociation
HPLC: High-performance liquid chromatography
HRP: Horseradish peroxidase
HSP90: Heat shock protein 90
IFNγ: Interferon γ
IgG: Immunoglobulin G
IKK: IκB kinase
IKKα: IκB kinase α
IKKβ: IκB kinase β
IKKγ: IκB kinase γ
IL-10: Interleukin-10
IL-12: Interleukin-12
IL-12p35: Interleukin-12 protein of 35 kDa
IL-12p40: Interleukin-12 protein of 40 kDa
IL-12p70: Interleukin-12 protein of 70 kDa
IL-2: Interleukin-2
IL-4: Interleukin-4
Il12a: Gene of interleukin-12 subunit α
IP_3_: Inositol 1,4,5-trisphosphate
ITAM: Immunoreceptor tyrosine-based activation motif
IκB: Inhibitor of NFκB
IκBα: Inhibitor of NFκB subunit α
IκBβ: Inhibitor of NFκB subunit β
IκBε: Inhibitor of NFκB subunit ε
JAK1: Janus kinase 1
KLF2: Kruppel-like transcription factor 2
LAL: Limulus amebocyte lysate
LC/MS: Liquid chromatography-mass spectrometry
LC–MS/MS: Liquid chromatography followed by tandem mass spectrometry
M1: Classically activated macrophage
M2: Alternatively activated macrophage
MALT1: Mucosa-associated lymphoid tissue lymphoma translocation protein 1
MAMP: Microbe associated molecular pattern
MAPK: Mitogen-activated protein kinase
mBMDC: Murine bone marrow-derived dendritic cell
MBR: Mannose-binding receptor
MDSC: Myeloid-derived suppressor cell
MFI: Mean fluorescence intensity
MS/MS: Tandem mass spectrometry
MS1: First run of MS in MS/MS
MS2: Second run of MS in MS/MS
MWCO: Molecular weight cut-off
NEMO: NFκB essential modulator
NFKB1: Nuclear factor kappa B subunit 1
NFκB: Nuclear factor kappa light chain-enhancer of activated B cells
NIK: NFκB inducing kinase
NLGP: Neem leaf glycoprotein
NLP: Neem leaf preparation
NLR: Nucleotide oligomerization domain (NOD)-like receptor
NLS: Nuclear localization signal
Opti-MEM: Serum reduced, minimum essential medium
p35: Protein of 35 kDa
p40: Protein of 40 kDa
p50: Protein of 50 kDa
p65: Protein of 65 kDa
p105: Protein of 105 kDa
PAGE: Polyacrylamide gel electrophoresis
PAMP: Pathogen associated molecular pattern
pAPC: Professional antigen presenting cell
PBS: Phosphate buffered saline
PCR: Polymerase chain reaction
PIP_2_: Phosphatidylinositol 4,5-bisphosphate
PKCδ: Protein kinase C δ
PLCγ: Phospholipase C γ
PMSF: Phenylmethylsulfonyl fluoride
pPKCδ: Phosphorylated protein kinase C δ
PRR: Pattern recognition receptor
PSM: Peptide spectrum match
pSTAT3: Phosphorylated-signal transducer and activator of transcription 3
PTGS: Post-transcriptional gene silencing
PTM: Post-translational modification
PVDF: Polyvinylidene difluoride
Raf-1: Rapidly accelerated fibrosarcoma protein kinase 1
RCSB PDB: Research collaboratory for structural bioinformatics-Protein data bank
RIP2: Receptor-interacting serine/threonine-protein kinase 2
RIPA: Radioimmunoprecipitation assay
RLR: RIG-I-like receptors
rmGM-CSF: Recombinant mouse-granulocyte macrophage-colony stimulating factor
rmIL-4: Recombinant mouse-interleukin-4
RMSD: Root mean square deviation
RNAi: RNA interference
ROX: 6-Carboxyl-X-Rhodamine
RPMI 1640: Roswell park memorial institute 1640
RT-PCR: Reverse transcription followed by polymerase chain reaction
RunX3: Runt-related transcription factor 3
SDS: Sodium dodecyl sulfate
siRNA: Small interfering RNA
SOCS3: Suppressor of cytokine signaling 3
Src kinase: Sarcoma kinase
ssDNA: Single stranded DNA
STAT3: Signal transducer and activator of transcription 3
STRING: Search tool for the retrieval of interacting genes/proteins
Syk: Spleen tyrosine kinase
TAD: Transcription activation domain
TAK1: TGFβ-activated kinase-1
TAM: Tumor-associated macrophage
T-bet: T-box expressed in T cells
Tbr2: T-box brain protein 2
Tbx21: T-Box transcription factor 21
T_c_: Cytotoxic T lymphocyte
TCM: Tumor conditioning media
TCR: T cell receptor
Tec kinase: Tec protein Tyrosine kinase
TFBM: Transcription factor binding motif
T_h_: Helper T lymphocyte
TLR: Toll-like receptor
TMB: 3,3′,5,5′-Tetramethylbenzidine
TRAF2: Tumor necrosis factor (TNF) receptor associated factor-2
TRAF6: Tumor necrosis factor (TNF) receptor associated factor-6
TRANSFAC: TRANScription FACtor database
T_reg_: Regulatory T lymphocyte
Ubc13: Ubiquitin-conjugating enzyme 13
Uev1A: Ubiquitin-conjugating enzyme variant 1A
