# Supplementary material for: Neem leaf glycoprotein binding to Dectin-1 receptors on dendritic cell induces type-1 immunity through CARD9 mediated intracellular signal to NFκB
Source: Cell Commun Signal. 2024 Apr 23;22:237. doi: 10.1186/s12964-024-01576-z (PMC11036628; doi:10.1186/s12964-024-01576-z)
Supplement: Supplementary file 1 — Supplementary Material 1. [file 12964_2024_1576_MOESM1_ESM.docx]

**Supplementary Information**

**Neem leaf glycoprotein binding to Dectin-1 receptors on dendritic cell induces type-1 immunity through CARD9 mediated intracellular signal to NFκB**

Nilanjan Ganguly^1^, Tapasi Das^1^, Avishek Bhuniya^1^, Ipsita Guha^1^, Mohona Chakravarti^1^, Sukanya Dhar^1^, Anirban Sarkar^1^, Saurav Bera^1^, Jesmita Dhar^2^, Shayani Dasgupta^1^, Akata Saha^1^, Tithi Ghosh^1^, Juhina Das^1^, Ugir Hossain Sk^3^, Saptak Banerjee^1^, Subrata Laskar^4^, Anamika Bose^1,5*^ and Rathindranath Baral^1^*

^1^Department of Immunoregulation and Immunodiagnostics, Chittaranjan National Cancer Institute, 37, S. P. Mukherjee Road, Kolkata, West Bengal, 700026, India

^2^Jubilant Biosys Limtied, 96, Digital Park Rd, Yesvantpur Industrial Suburb, Bengaluru, Karnataka, 560022, India

^3^Department of Clinical and Translational Research, Chittaranjan National Cancer Institute, 37, S. P. Mukherjee Road, Kolkata, West Bengal, 700026, India

^4^Department of Chemistry, University of Burdwan, Burdwan, West Bengal, 713104, India

5Department of Pharmaceutical Technology-Biotechnology, National Institute of Pharmaceutical Education and Research (NIPER),-S.A.S. Nagar, Mohali, Punjab, 160062, India

***Correspondence to:**

Rathindranath Baral, Ph.D

Department of Immunoregulation and Immunodiagnostics

Chittaranjan National Cancer Institute

37, S.P. Mukherjee Road, Kolkata – 700026

Telephone: 91-033-2476-5101 ext. 334

Email: [baralrathin@hotmail.com](mailto:baralrathin@hotmail.com)

**Supplementary Figures 1-3 (Figures S1-S3)**

**Supplementary Tables 1-6 (Tables S1-S6)**


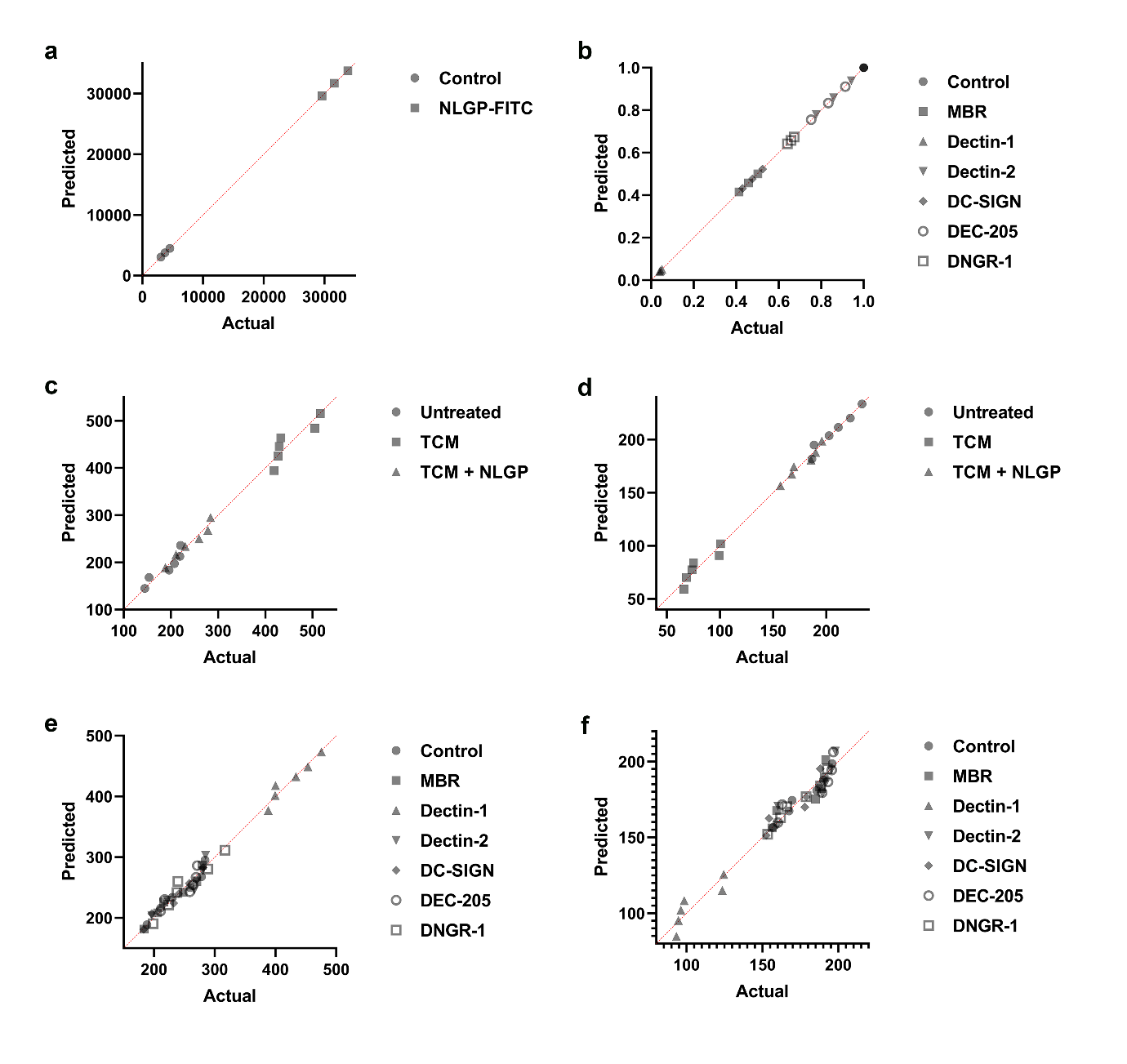


**Fig. S1 ǀ Normal QQ plots obtained by testing the Gaussian distribution of the data for checking NLGP-mBMDC interactions and those on obstructing C-type lectin receptors.** Normal distribution of the numerical data from experimental replicates of: **a** Figure 2b was determined by Shapiro-Wilk test. **b** Figure 2e was determined by Shapiro-Wilk test. **c** Figure 2i (left) was determined by Kolmogorov-Smirnov test. **d** Figure 2i (right) was determined by Kolmogorov-Smirnov test. **e** Figure 2l (left) was determined by Kolmogorov-Smirnov test. **f** Figure 2l (right) was determined by Kolmogorov-Smirnov test.


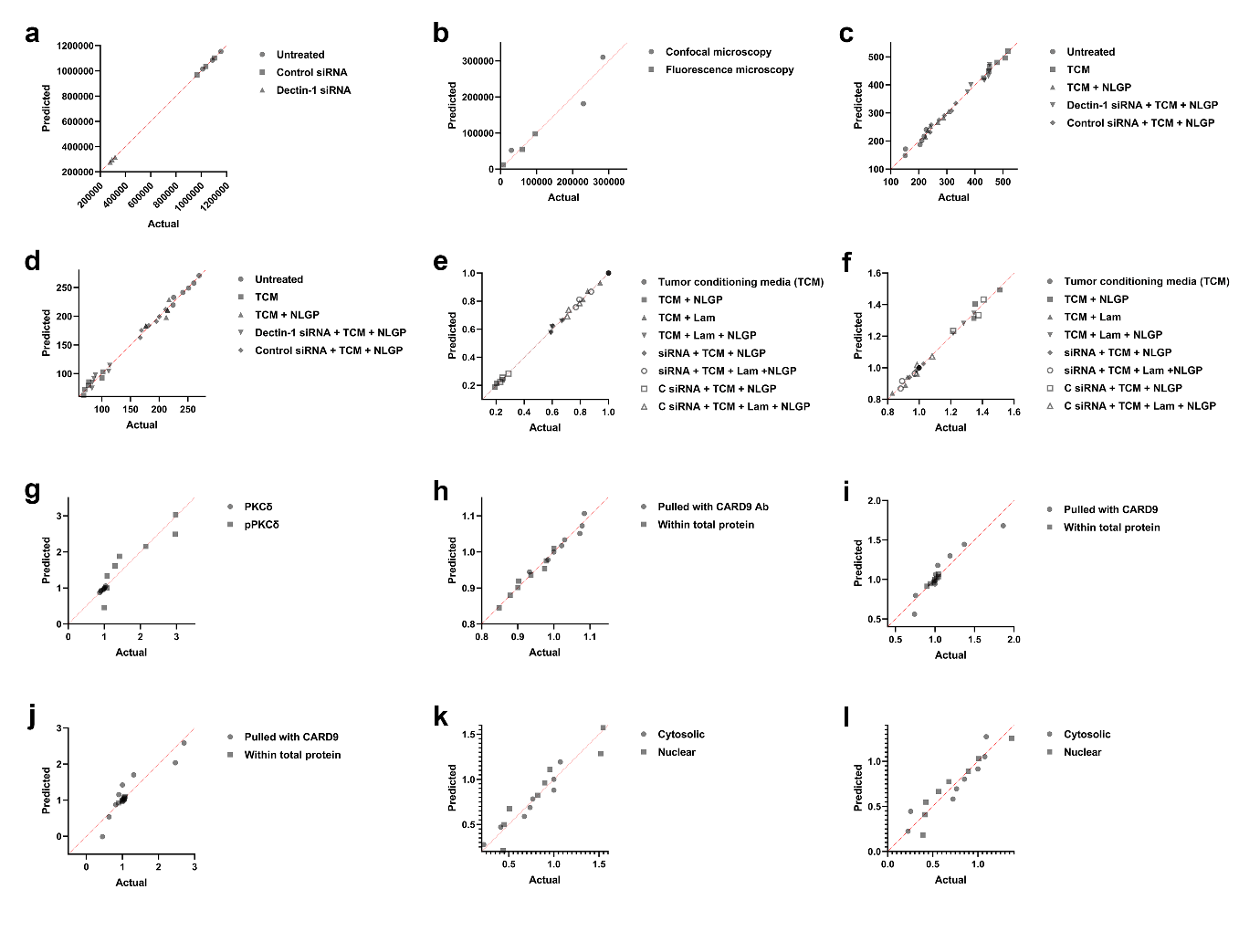


**Fig. S2 ǀ Normal QQ plots obtained by testing the Gaussian distribution of the data for checking NLGP-mBMDC interactions following RNAi based Dectin-1 knockdown and exploring the second messengers of the signal ensuing from Dectin-1 upon NLGP-binding.** Normal distribution of the numerical data from experimental replicates of: **a** Figure 3a was determined by Shapiro-Wilk test. **b** Figure 3c was determined by Shapiro-Wilk test. **c** Figure 3f (above) was determined by Kolmogorov-Smirnov test. **d** Figure 3f (below) was determined by Kolmogorov-Smirnov test. **e** Figure 5a (left) was determined by Shapiro-Wilk test. **f** Figure 5a (right) was determined by Shapiro-Wilk test. **g** Figure 5c was determined by Kolmogorov-Smirnov test. **h** Figure 5d (upper right) was determined by Kolmogorov-Smirnov test. **i** Figure 5d (lower left) was determined by Kolmogorov-Smirnov test. **j** Figure 5d (lower right) was determined by Kolmogorov-Smirnov test. **k** Figure 5e (lower left) was determined by Kolmogorov-Smirnov test. **l** Figure 5e (lower right) was determined by Kolmogorov-Smirnov test.


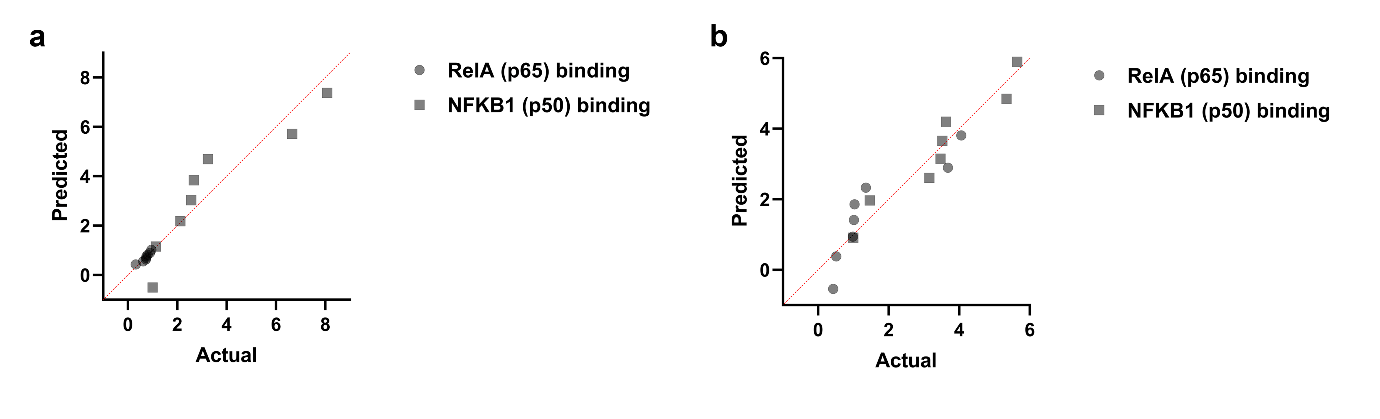


**Fig. S3 ǀ Normal QQ plots obtained by testing the Gaussian distribution of the data for binding of NFκB subunits (RelA/p65 and NFKB1/p50) obtained from chromatin immunoprecipitation assays.** Normal distribution of the numerical data from experimental replicates of: **a** Figure 7B was determined by D’Agostino & Pearson test. **b** Figure 7C was determined by D’Agostino & Pearson test.

**Table S1 ǀ NLGP derived peptides obtained by database search**

| Peptide Sequence | Peptide score (-10 log_10_p) | m/z | Mass (Da) | RT (mins) | Length | Accession |
| --- | --- | --- | --- | --- | --- | --- |
| ILDEALAGDNVGLLLR | 83.83 | 561.3119 | 1680.9409 | 27.20 | 16 | P46280 |
| VAPEEHPVLLTEAPLNPK | 81.07 | 652.0245 | 1953.0570 | 23.41 | 18 | P46258 |
| EAYPGDVFYLHSR | 76.94 | 518.5828 | 1552.7310 | 24.29 | 13 | B0Z4W6 |
| EITLGFVDLLR | 58.21 | 638.3677 | 1274.7234 | 32.86 | 11 | P93998 |

Abbreviations: m/z – mass-to-charge ratio, Da – Daltons, RT – Retention Time, mins – minutes.

**Table S2 ǀ NLGP derived peptides obtained by *de novo* sequencing (average local confidence = 96)**

| Peptide Sequence | Length | m/z | Mass (Da) | RT (mins) | ALC (%) | De novo Score |
| --- | --- | --- | --- | --- | --- | --- |
| VDLLNQELEFLK | 12 | 730.9017 | 1459.7922 | 30.67 | 97 | 97 |
| LALDLELATYR | 11 | 639.3571 | 1276.7026 | 28.4 | 97 | 97 |
| LSNLEAQLTDVR | 12 | 679.8657 | 1357.7202 | 23.88 | 97 | 97 |
| LQVRVATVSLPR | 12 | 446.9443 | 1337.8142 | 22.01 | 97 | 97 |
| MTLDDFR | 7 | 449.2095 | 896.4062 | 24.48 | 96 | 96 |

Abbreviations: m/z – mass-to-charge ratio, Da – Daltons, RT – Retention Time, mins – minutes, ALC – Average Local Confidence.

**Table S3 ǀ Fluorescence signal quantifications from micrographs, showing binding of NLGP-FITC to dendritic cells**

|  | Mean Grey Value (FITC) of cells (8 bit) | Mean Integrated Density | Mean CTCF/Mean Fluorescence Intensity/MFI |
| --- | --- | --- | --- |
| Control | 7.227 | 3728.143 | 3711.546 |
| NLGP-FITC | 28.219 | 31653.100 | 31602.760 |

The ‘Mean Grey Values’ of the ‘FITC’ channel of the cells are determined by dividing the ‘Total Integrated Density’ of the cells, by the ‘Total Area’ of those cells in each field.

Abbreviations: FITC – Fluorescin isothiocyanate, NLGP-FITC – Neem Leaf Glycoprotein molecules chemically attached to Fluorescin isothiocyanate, CTCF – Corrected Total Cell Fluorescence, MFI: Mean Fluorescence Intensity.

**Table S4 ǀ Fluorescence signal quantifications from micrographs, showing binding of NLGP-FITC to dendritic cells on blocking the mentioned cell surface glycoprotein receptors**

|  | Mean Grey Value (FITC) of cells (8 bit) | Mean Integrated Density | Mean CTCF/Mean Fluorescence Intensity/MFI |
| --- | --- | --- | --- |
| Control | 38.599 | 60236.290 | 58190.290 |
| MBR | 39.620 | 41693.730 | 40478.130 |
| Dectin-1 | 4.965 | 3904.875 | 3903.103 |
| Dectin-2 | 47.992 | 75952.800 | 75819.350 |
| DC-SIGN | 28.926 | 42814.570 | 42170.980 |
| DEC-205 | 37.109 | 74738.330 | 73651.590 |
| DNGR-1 | 53.609 | 90224.570 | 88321.470 |

The ‘Mean Grey Values’ of the ‘FITC’ channel of the cells are determined by dividing the ‘Total Integrated Density’ of the cells, by the ‘Total Area’ of those cells in each field.

Abbreviations: FITC – Fluorescin isothiocyanate, CTCF – Corrected Total Cell Fluorescence, MFI: Mean Fluorescence Intensity, MBR – Mannose Binding Receptor.

**Table S5 ǀ Fluorescence signal quantifications from micrographs, showing binding of NLGP-FITC to dendritic cells on downregulating Dectin-1 expression by siRNA treatment**

|  | Mean Grey Value (FITC) of cells (8 bit) | Mean Integrated Density | Mean CTCF/Mean Fluorescence Intensity/MFI |
| --- | --- | --- | --- |
| Confocal Microscopy | | | |
| Untreated | 30.196 | 294392.700 | 283812.300 |
| Control siRNA | 14.315 | 243305.000 | 229283.300 |
| Dectin-1 siRNA | 4.0827 | 39445.330 | 30595.900 |
| Widefield Fluorescence Microscopy | | | |
| Untreated | 35.502 | 97072.290 | 95955.160 |
| Control siRNA | 26.825 | 61042.133 | 60564.318 |
| Dectin-1 siRNA | 6.421 | 7663.000 | 7663.000 |

The ‘Mean Grey Values’ of the ‘FITC’ channel of the cells are determined by dividing the ‘Total Integrated Density’ of the cells, by the ‘Total Area’ of those cells in each field.

Abbreviations: FITC – Fluorescin isothiocyanate, CTCF – Corrected Total Cell Fluorescence, MFI: Mean Fluorescence Intensity.

**Table S6 ǀ Oligonucleotides used in the study**

| Primers for PCR and qPCR | | | |
| --- | --- | --- | --- |
| Gene  (*Mus musculus*) | Forward (5’→3’) | | Reverse (5’→3’) |
| IL-10 | GTACAGCCGGGAAGACAATA | | GGCAACCCAAGTAACCCTTA |
| IL-12A | CCTAAACCACCTCAGTTTGG | | GATCGATGTCTTCAGCAGTG |
| β actin | CAACCGTGAAAAGATGACCC | | ATGAGGTAGTCTGTCAGGTC |
| Primers for gene operator sequence (TFBM) amplifications in chromatin immunoprecipitation | | | |
| Operator (TFBM)  (*Mus musculus*) | Forward (5’→3’) | | Reverse (5’→3’) |
| p65-TFBM on IL-10 gene | GTGAGGTCTGAAGAAAATCA | | TAATTGTTTCCTCCACTCAA |
| p50-TFBM on IL-10 gene | CTCTAGGCGAATGTTCTTC | | GTTGAAGGATGGAGATGTTA |
| p65-TFBM on IL-12A gene | AAGCTTCGAACTCTCACAC | | TCCATTCAAGATGGAGAAC |
| p50-TFBM on IL-12A gene | TGTTGTCCACAATCTCTCG | | TGCATTCTGAGCTAGTTTTG |
| Oligonucleotide sequences for *Mus musculus* Dectin-1 siRNA synthesis | | | |
| Sense (5’→3’) | | Antisense (5’→3’) | |
| AATTCTCTGATCCCCTGGGCCCCTGTCTC | | AAGGCCCAGGGGATCAGAGAACCTGTCTC | |
