# Supplementary material for: Neem leaf glycoprotein binding to Dectin-1 receptors on dendritic cell induces type-1 immunity through CARD9 mediated intracellular signal to NFκB
Source: Cell Commun Signal. 2024 Apr 23;22:237. doi: 10.1186/s12964-024-01576-z (PMC11036628; doi:10.1186/s12964-024-01576-z)
Supplement: Supplementary file 2 — Supplementary Material 2. [file 12964_2024_1576_MOESM2_ESM.docx]

**Key Resources Table**

| **REAGENT or RESOURCE** | **SOURCE** | **IDENTIFIER** |
| --- | --- | --- |
| **Antibodies** | | |
| Anti-1, 3 beta glucan antibody [2G8] | Abcam | Cat# ab233743; RRID:AB_2923478 |
| Anti-CLEC7A antibody | MyBioSource | Cat# MBS837653 |
| Anti-IL-10 antibody | BioLegend | Cat# 505001; RRID:AB_315355 |
| Anti-IL-12 p35 antibody | R&D Systems | Cat# MAB6688; RRID:AB_10890933 |
| Anti-Dectin-2/CLEC6A alpha Isoform antibody | R&D systems | Cat# AF1525; RRID:AB_2229601 |
| Anti-DC-SIGN/CD209 antibody | R&D systems | Cat# MAB83451 |
| Anti-DEC-205/CD205 antibody | R&D systems | Cat# AF5975; RRID:AB_1964561 |
| Anti-CLEC9A antibody | R&D systems | Cat# AF6776; RRID:AB_10890771 |
| Anti-PKC delta antibody (G-9) | Santa Cruz Biotechnology | Cat# sc-8402; RRID:AB_628145 |
| Anti-p-PKC delta antibody (A-8) | Santa Cruz Biotechnology | Cat# sc-377560 |
| Anti-CARD9 antibody [N1N3] | GeneTex | Cat# GTX110789; RRID:AB_1949833 |
| Anti-BCL10 antibody | BioLegend | Cat# 684702; RRID:AB_2572170 |
| Anti-MALT1 mouse antibody | Elabscience | Cat# E-AB-60635 |
| Anti-NF-kB p65 antibody | Abcam | Cat# ab7970; RRID:AB_306184 |
| Anti-NFκB p50 antibody (E-10) | Santa Cruz Biotechnology | Cat# sc-8414; RRID:AB_628015 |
| Anti-beta Actin antibody (C4) | Santa Cruz Biotechnology | Cat# sc-47778; RRID:AB_626632 |
| Anti-Histone H3 (C-terminus) antibody | BioLegend | Cat# 819412; RRID:AB_2820128 |
| FITC-conjugated Goat anti-Rat IgG | Sigma-Aldrich | Cat# F6258; RRID:AB_259695 |
| HRP-conjugated Goat anti-Mouse IgG | Sigma-Aldrich | Cat# 12-349; RRID:AB_390192 |
| HRP-conjugated Goat anti-Rat IgG | Sigma-Aldrich | Cat# AP136P; RRID:AB_11214444 |
| HRP-conjugated Goat anti-Rabbit IgG | Sigma-Aldrich | Cat# 12-348; RRID:AB_390191 |
| **Chemicals, peptides, and recombinant proteins** | | |
| RPMI 1640 Medium, GlutaMAX™ Supplement | Gibco | Cat# 72400047 |
| DMEM, high glucose | Gibco | Cat# 11995065 |
| Opti-MEM™, Reduced Serum Medium | Gibco | Cat# 31985070 |
| Fetal Bovine Serum | HiMedia Laboratories | Cat# RM10409 |
| L-Glutamine-Penicillin-Streptomycin Solution | HiMedia Laboratories | Cat# A007 |
| Trypsin (0.25%), phenol red | Gibco | Cat# 15050057 |
| EDTA Disodium Salt Dihydrate | SRL | Cat# 43272 |
| Phosphate Buffered Saline, pH 7.2 10X | HiMedia Laboratories | Cat# TL1032 |
| 10X RBC Lysis Buffer | HiMedia Laboratories | Cat# R075 |
| Lipofectamine™ 2000 Transfection Reagent | Thermo Fisher Scientific | Cat# 11668019 |
| eBioscience™ Brefeldin A Solution (1000X) | Invitrogen | Cat# 00-4506-51 |
| Cytofix/Cytoperm™ Plus Fixation/Permeabilization Solution Kit | BD Biosciences | Cat# 554715 |
| Paraformaldehyde Solution, 4% | HiMedia Laboratories | Cat# TCL119 |
| Fluoroshield™ with DAPI | Sigma-Aldrich | Cat# F6057 |
| Fluorescein isothiocyanate isomer I | Sigma-Aldrich | Cat# F7250 |
| Dimethyl sulfoxide | Sigma-Aldrich | Cat# 276855 |
| Triethylamine | Sigma-Aldrich | Cat# 471283 |
| Methanol | Sigma-Aldrich | Cat# 322415 |
| TRIzol™ Reagent | Invitrogen | Cat# 15596018 |
| Proteinase K | Thermo Fisher Scientific | Cat# EO0491 |
| Phenol | Sigma-Aldrich | Cat# 33517 |
| Chloroform | Sigma-Aldrich | Cat# 288306 |
| Ethanol | Sigma-Aldrich | Cat# 02870 |
| Takyon™ Low ROX SYBR 2X MasterMix blue dTTP | Eurogentec | Cat# UF-LSMT-B0701 |
| GoTaq® Green Master Mix | Promega | Cat# M7122 |
| Silencer® Select Negative Control siRNA | Invitrogen | Cat# 4390843 |
| Agarose low EEO | SRL | Cat# 36601 |
| Ethidium bromide solution | Sigma-Aldrich | Cat# E1510 |
| Bovine Serum Albumin, BSA | SRL | Cat# 83803 |
| Bradford Reagent | Sigma-Aldrich | Cat# B6916 |
| OptEIA™ TMB Substrate Reagent Set | BD Biosciences | Cat# 555214 |
| Sulfuric acid | Sigma-Aldrich | Cat# 339741 |
| E-Toxate™ reagent from *Limulus polyphemus* | Sigma-Aldrich | Cat# E8779 |
| Halt™ Protease Inhibitor Cocktail (100X) | Thermo Fisher Scientific | Cat# 78429 |
| Bromophenol Blue | Sigma-Aldrich | Cat# B0126 |
| Tris base | Sigma-Aldrich | Cat# 10708976001 |
| Boric Acid | SRL | Cat# 80266 |
| Tris hydrochloride | Roche | Cat# 10812846001 |
| Potassium chloride | Sigma-Aldrich | Cat# P4504 |
| Sodium chloride | Sigma-Aldrich | Cat# S3014 |
| Magnesium chloride | Sigma-Aldrich | Cat# M8266 |
| HEPES (1 M) | Gibco | Cat# 15630080 |
| Sodium orthovanadate | Sigma-Aldrich | Cat# S6508 |
| Glycerol | SRL | Cat# 77453 |
| Glycine | Sigma-Aldrich | Cat# 410225 |
| Formaldehyde solution about 37% | Sigma-Aldrich | Cat# 1.04003 |
| Acetonitrile solution | Sigma-Aldrich | Cat# 900686 |
| PMSF | Roche | Cat# 10837091001 |
| Acrylamide 40% aq. solution | SRL | Cat# 29787 |
| N,N-Methylene Bisacrylamide | SRL | Cat# 38862 |
| Ammonium persulfate | Sigma-Aldrich | Cat# A7460 |
| N,N,N′,N′-Tetramethylethylenediamine | Sigma-Aldrich | Cat# T9281 |
| TWEEN® 20 | Sigma-Aldrich | Cat# P9416 |
| NP-40 Surfact-Amps™ | Thermo Fisher Scientific | Cat# 85124 |
| Pierce™ DTT (Dithiothreitol), No-Weigh™ | Thermo Fisher Scientific | Cat# A39255 |
| Sodium Deoxycholate Detergent | Thermo Fisher Scientific | Cat# 89904 |
| Sodium Dodecyl Sulfate | Thermo Fisher Scientific | Cat# 28364 |
| 2-Mercaptoethanol | Gibco | Cat# 21985023 |
| WesternBright ECL HRP substrate | Advansta | Cat# K-12045-D20 |
| Mannan from *Saccharomyces cerevisiae* | Sigma-Aldrich | Cat# M7504 |
| Laminarin, soluble beta-glucan from *Laminaria digitata* | InvivoGen | Cat code# tlrl-lam |
| Recombinant Mouse GM-CSF | Miltenyi Biotec | Cat# 130-095-746 |
| Recombinant Mouse IL-4 | Miltenyi Biotec | Cat# 130-097-757 |
| **Critical commercial assays** | | |
| SOLu-Trypsin Rapid Digestion Kit | Sigma-Aldrich | Cat# MSKT0002 |
| RevertAid First Strand cDNA Synthesis Kit | Thermo Fisher Scientific | Cat# K1622 |
| Silencer™ siRNA Construction Kit | Invitrogen | Cat# AM1620 |
| Chromatin Immunoprecipitation (ChIP) Assay Kit | Sigma-Aldrich | Cat# 17-295 |
| **Experimental models: Cell lines** | | |
| B16-F10 | ATCC | Cat# CRL-6475; RRID:CVCL_0159 |
| **Experimental models: Organisms/strains** | | |
| Mouse: C57BL/6J | The Jackson Laboratory | RRID: IMSR_JAX:000664 |
| **Oligonucleotides** | | |
| See Table S6 | N/A | N/A |
| **Software and algorithms** | | |
| PEAKS Studio (v11) | Bioinformatics Solutions Inc. | RRID:SCR_022841; https://www.bioinfor.com/peaks-studio/ |
| INFINITY CAPTURE Windows (v6.5.7) | Lumenera Corporation | https://www.lumenera.com/infinity-capture-windows.html |
| ZEN Microscopy Software (v3.7) | Zeiss | RRID:SCR_013672; https://www.zeiss.com/microscopy/en/products/software/zeiss-zen.html |
| Image Lab (v6.1) | Bio-Rad | RRID:SCR_014210; https://www.bio-rad.com/en-in/product/image-lab-software |
| Fiji (v1.52p) | Fiji | RRID:SCR_002285; https://fiji.sc/ |
| DataAssist (v3.01) | Applied Biosystems | RRID:SCR_014969; https://www.thermofisher.com/in/en/home/technical-resources/software-downloads/dataassist-software.html |
| BD CellQuest Pro (v5.2) | BD Biosciences | RRID:SCR_014489; https://www.bdbiosciences.com/content/dam/bdb/marketing-documents/14_cellquest_prosoft_acquisit.pdf |
| FlowJo (v10.7.2) | BD Biosciences | RRID:SCR_008520; https://www.flowjo.com/ |
| SoftMax Pro (v7.1) | Molecular Devices | RRID:SCR_014240; https://www.moleculardevices.com/products/microplate-readers/acquisition-and-analysis-software/softmax-pro-software |
| Open Babel GUI | Open Babel | RRID:SCR_014920;  https://openbabel.org/docs/current/GUI/GUI.html |
| AutoDock (v4.2.6) | Scripps Research | RRID:SCR_012746; https://autodock.scripps.edu/ |
| BIOVIA Discovery Studio Visualizer (v20.1.0.19295) | Dassault Systemes | RRID:SCR_015651; https://www.3ds.com/products-services/biovia/products/molecular-modeling-simulation/biovia-discovery-studio/ |
| ALGGEN-PROMO | BarcelonaTech | RRID:SCR_016926; https://alggen.lsi.upc.es/cgi-bin/promo_v3/promo/promoinit.cgi?dirDB=TF_8.3https://alggen.lsi.upc.es/cgi-bin/promo_v3/promo/promoinit.cgi?dirDB=TF_8.3 |
| BioEdit (v7.2.5) | Thomas Hall, Microbiology, NCSU | RRID:SCR_007361; http://www.mbio.ncsu.edu/BioEdit/bioedit.html |
| Prism (v8.4.2) | GraphPad | RRID:SCR_002798; https://www.graphpad.com/ |
| **Other** | | |
| SnakeSkin™ Dialysis Tubing | Thermo Fisher Scientific | Cat# 88245 |
| Amicon® Ultra-4 Centrifugal Filter | Millipore | Cat# UFC801024 |
| Millex-GP Syringe-Filter (0.22 µm) | Millipore | Cat# SLGP033RS |
| µBondapak C18 Prep Column | Waters | Cat# WAT084176 |
| Protein G Sepharose® 4 Fast Flow | Sigma-Aldrich | Cat# GE17-0618-01 |
| WesternBright PVDF‑FL | Advansta | Cat# L-08001-010 |
| UniProtKB/Swiss-Prot | UniProt | RRID:SCR_021164; https://www.expasy.org/resources/uniprotkb-swiss-prot |
| RCSB PDB | RCSB | RRID:SCR_012820; https://www.rcsb.org/ |
| CHEBI | EMBL-EBI | RRID:SCR_002088; https://www.ebi.ac.uk/chebi/ |
| Eukaryotic Promoter Database | Swiss Institute of Bioinformatics | RRID:SCR_002132; https://epd.epfl.ch/EPD_database.php |
