# Supplementary material for: Neem leaf glycoprotein binding to Dectin-1 receptors on dendritic cell induces type-1 immunity through CARD9 mediated intracellular signal to NFκB
Source: Cell Commun Signal. 2024 Apr 23;22:237. doi: 10.1186/s12964-024-01576-z (PMC11036628; doi:10.1186/s12964-024-01576-z)
Supplement: Supplementary file 3 — Supplementary Material 3. [file 12964_2024_1576_MOESM3_ESM.docx]

**Description of supplementary video files in additional files 4-8**

Additional Supplementary Information are included as five supplementary video files in Additional files 4-8. Following are descriptions of each**:**

**Additional file 4 ǀ A video of a rotating complex of three small molecules (short β-D-Glucan chains, having 1-3, 1-6; 1-3 and 1-4 linkages) docked on to murine Dectin-1 dimer.** Surface view of dimeric murine Dectin-1 (2BPE) procured from RCSB PDB, along with the docked β-D-Glucan flexible short chains is rotated to show the binding cavities of the chains on the rigid macromolecule. Later, representation of the same Dectin-1 dimer surface is changed from solid single color (blue or cyan) to hydrophobicity-based regions (hydrophobicity scale shown in Fig. 6d). Even later, the secondary structure-based ribbon representation of this Dectin-1 dimer is revealed from within the surface and the local interactions of the β-D-Glucan short chains are shown, with bound Dectin-1-amino acids. The immunoreceptor tyrosine-based activation motifs (ITAMs) on the cytosolic face of Dectin-1 dimer are represented in black within the rainbow-colored ribbon representation of the dimer and are shown by the mouse pointer, nearly at the end of the video.

**Additional file 5 ǀ A video of the images of mBMDCs stained or treated with NLGP-FITC forming a Z-stack.** Fluorescence micrographs of murine bone marrow-derived dendritic cells (mBMDCs), incubated with NLGP-FITC for 1 hr, washed and fixed (nuclei stained with DAPI). These two-dimensional micrographs of a field were captured along successive layers of depth through Z-axis, each separated from the previous and next ones by a specific-thickness-interval, producing a stack (Z-stack) of images. In the video, individual images of the Z-stack are shown gradually from upper to lower surfaces along depth (scale bar at bottom right).

**Additional file 6 ǀ A video of rotation of the surface-view of NLGP-FITC treated mBMDCs.** Cellular surfaces of the mBMDCs incubated with NLGP-FITC for 1 hr, on three-dimensional reconstruction of the Z-stack as a surface-view. Reference frame with the axes is shown at the top left corner of the field.

**Additional file 7 ǀ A video of rotation of the volume-view of NLGP-FITC treated mBMDCs.** Emission of FITC-fluorescence from the mBMDCs incubated with NLGP-FITC for 1 hr, reconstructed in three-dimensions from the Z-stack as the volume-view of the cells. Reference frame is shown at the top left corner of the field.

**Additional file 8 ǀ A video showing a longitudinal section through the volume-view of mBMDCs exposing interiors of the NLGP-FITC treated cells.** A longitudinal section parallel to Y-Z plane of the volume-view of the mBMDCs incubated with NLGP-FITC for 1 hr, reveals the FITC fluorescence signals from the interiors of the cells. This revealed intracellular zone is visualized upon rotation. Reference frame is shown at the top-left corner of the field.
