# Supplementary material for: Neem leaf glycoprotein binding to Dectin-1 receptors on dendritic cell induces type-1 immunity through CARD9 mediated intracellular signal to NFκB
Source: Cell Commun Signal. 2024 Apr 23;22:237. doi: 10.1186/s12964-024-01576-z (PMC11036628; doi:10.1186/s12964-024-01576-z)
Supplement: Supplementary file 9 — Supplementary Material 9. [file 12964_2024_1576_MOESM9_ESM.pdf]

Uncropped images of gels and blots

Agarose gels from Figures 2 and 3:

Fig. 2g

Transcript-quantifications of cytokines IL-10 and IL-12A

(Effects of TCM and NLGP on DCs)

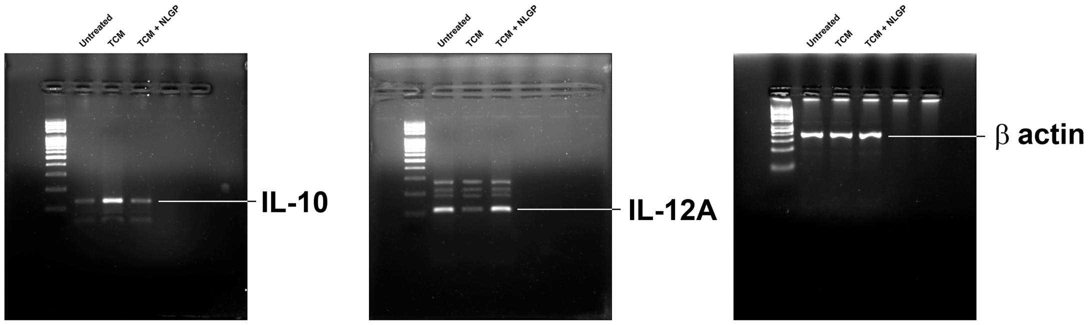

Fig. 2j

Transcript-quantifications of cytokines IL-10 and IL-12A (On blocking C-type lectins on tumor-conditioned NLGP-treated DCs)

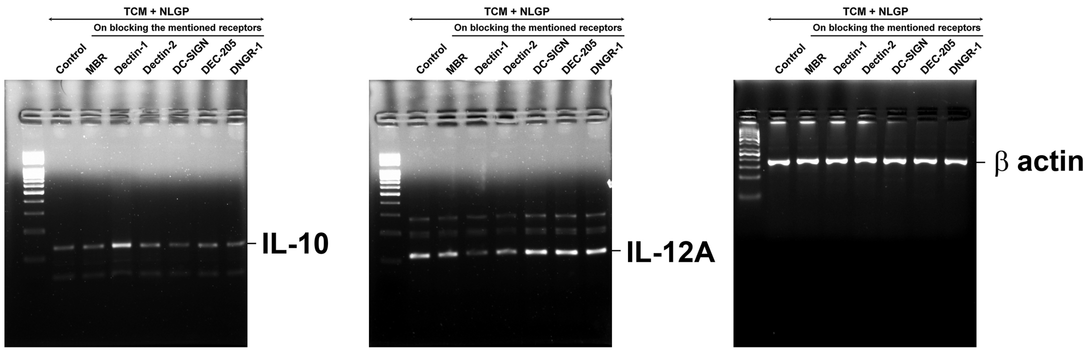

Fig. 3d

Transcript-quantifications of cytokines IL-10 and IL-12A

(Upon knocking down Dectin-1 through RNAi)

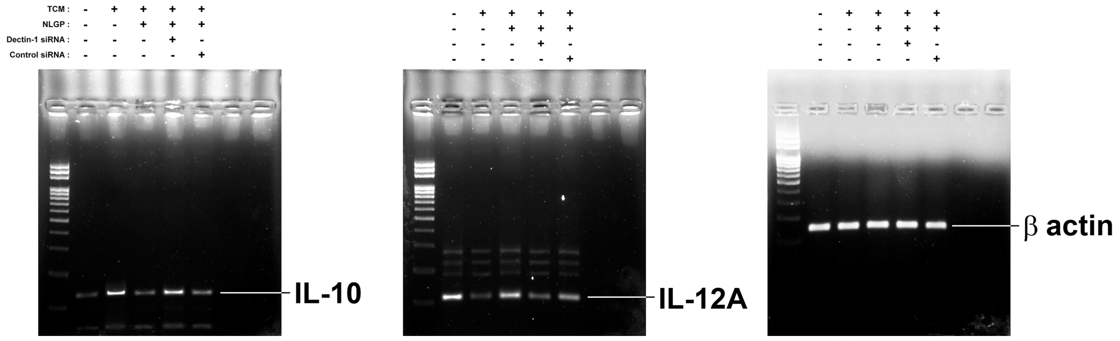

Western blots from Figure 5:

Images of the ladders on the blot membranes were captured through a separate colorimetric channel, other than the bands and both were superimposed later. Since, the bands were quantified

and cropped out of the original blot images (without ladders), both sets (with and without ladders) of uncropped images are given below:

Western blot images without ladders:

Fig. 5c

Phosphorylation of PKCδ

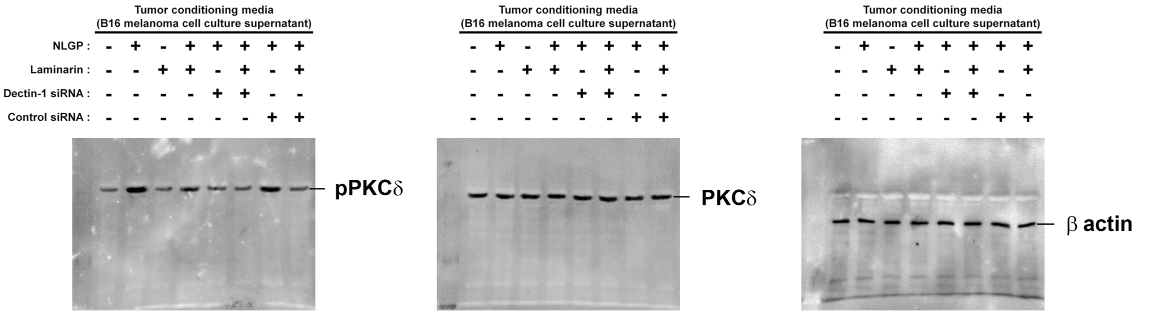

Fig. 5d
Co-immunoprecipitation

Samples pulled with anti-CARD9 antibody

Total cellular protein samples

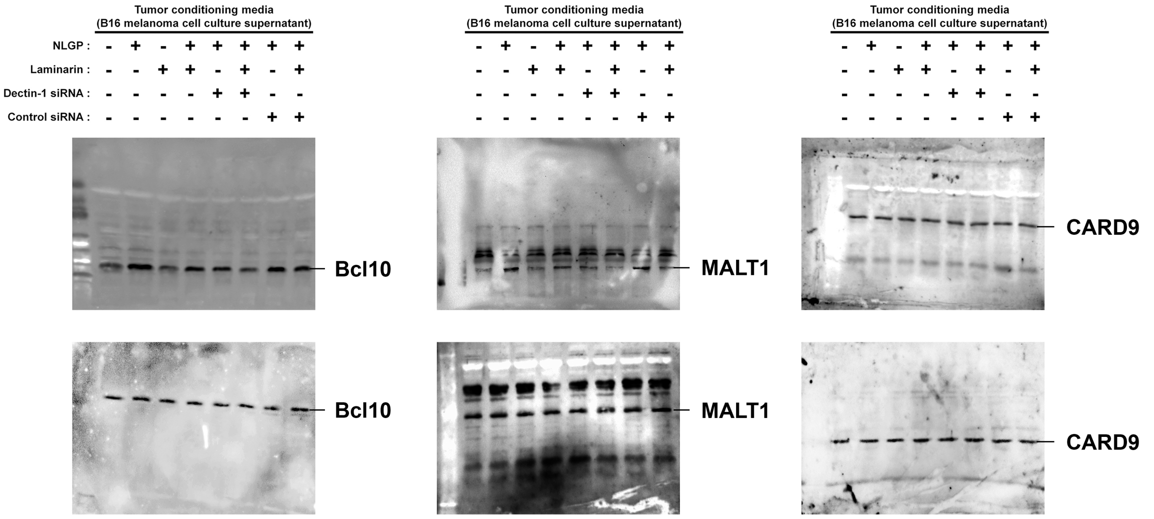

Fig. 5e

Assesing nuclear translocation of NFκB

Within cytosolic protein fractions

Within nuclear protein fractions

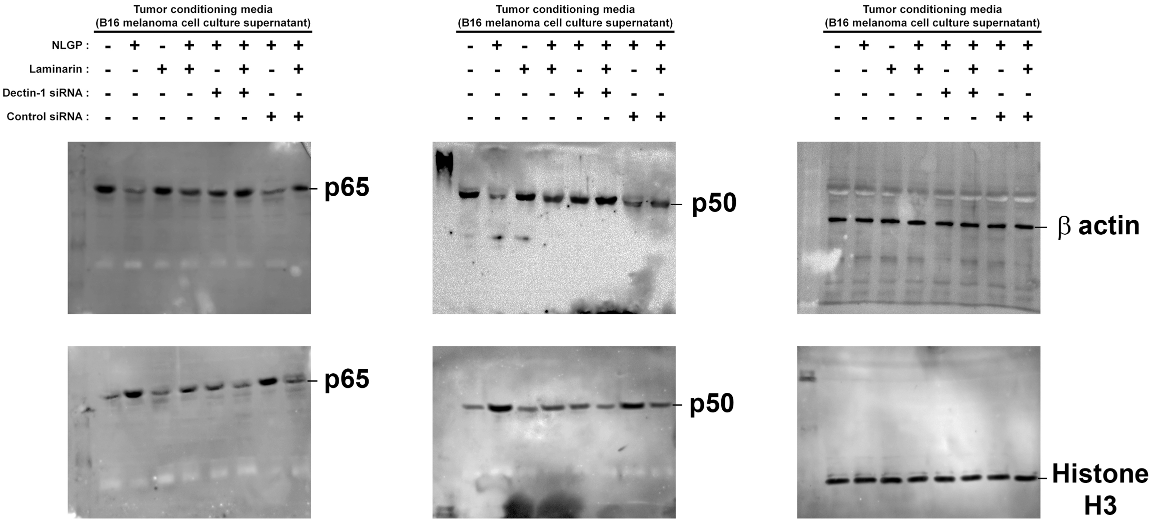

Western blot images with ladders:

Fig. 5c

Phosphorylation of PKCδ

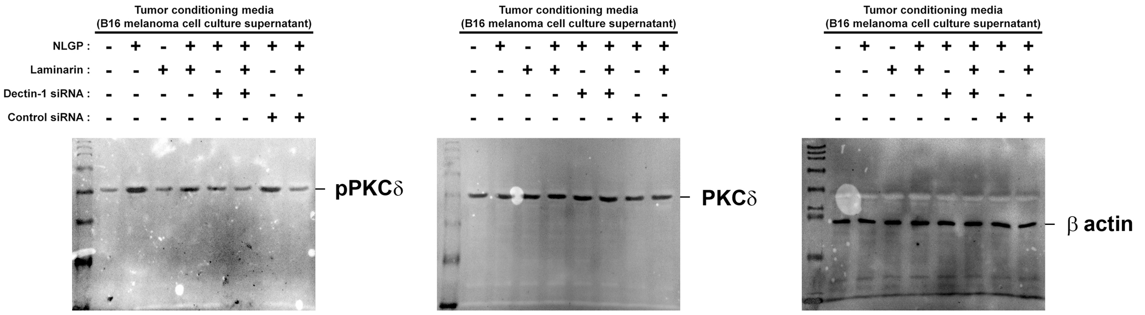

Fig. 5d
Co-immunoprecipitation

Samples pulled with anti-CARD9 antibody

Total cellular protein samples

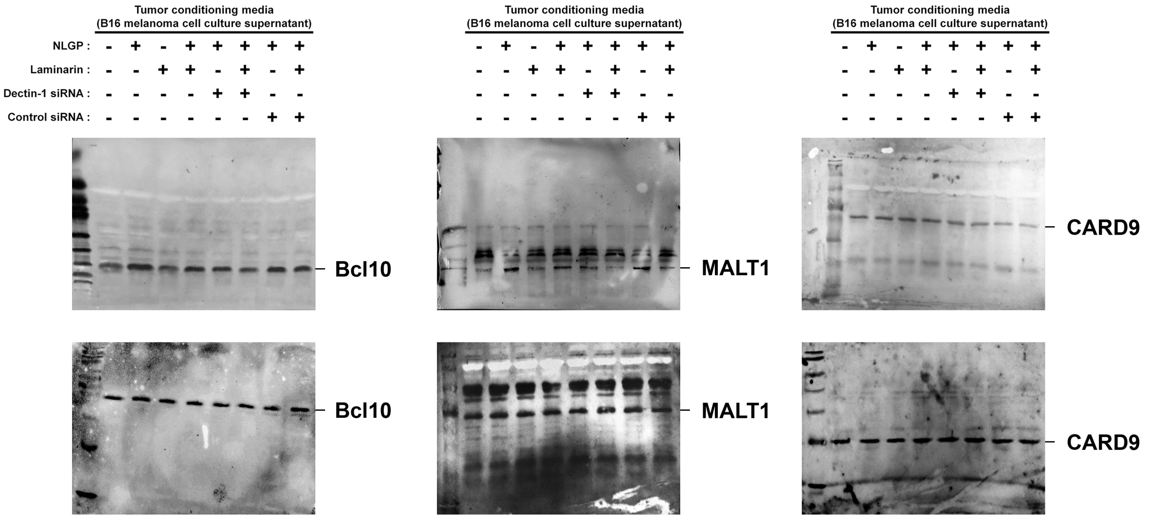

Fig. 5e

Assesing nuclear translocation of NFκB

Within cytosolic protein fractions

Within nuclear protein fractions

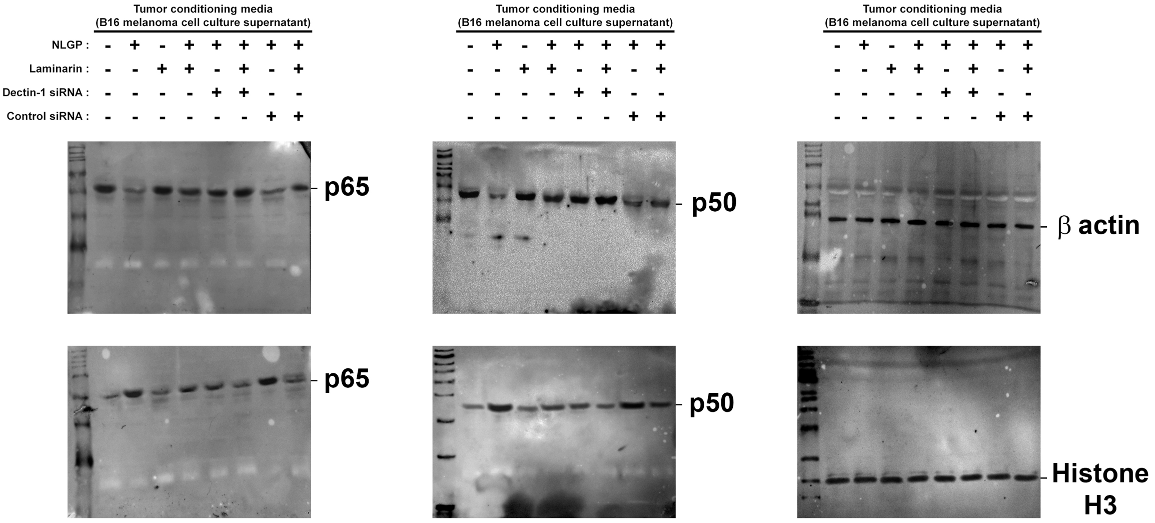

Agarose gels electrophoresis after Chromatin immunoprecipitation from Figure 7:

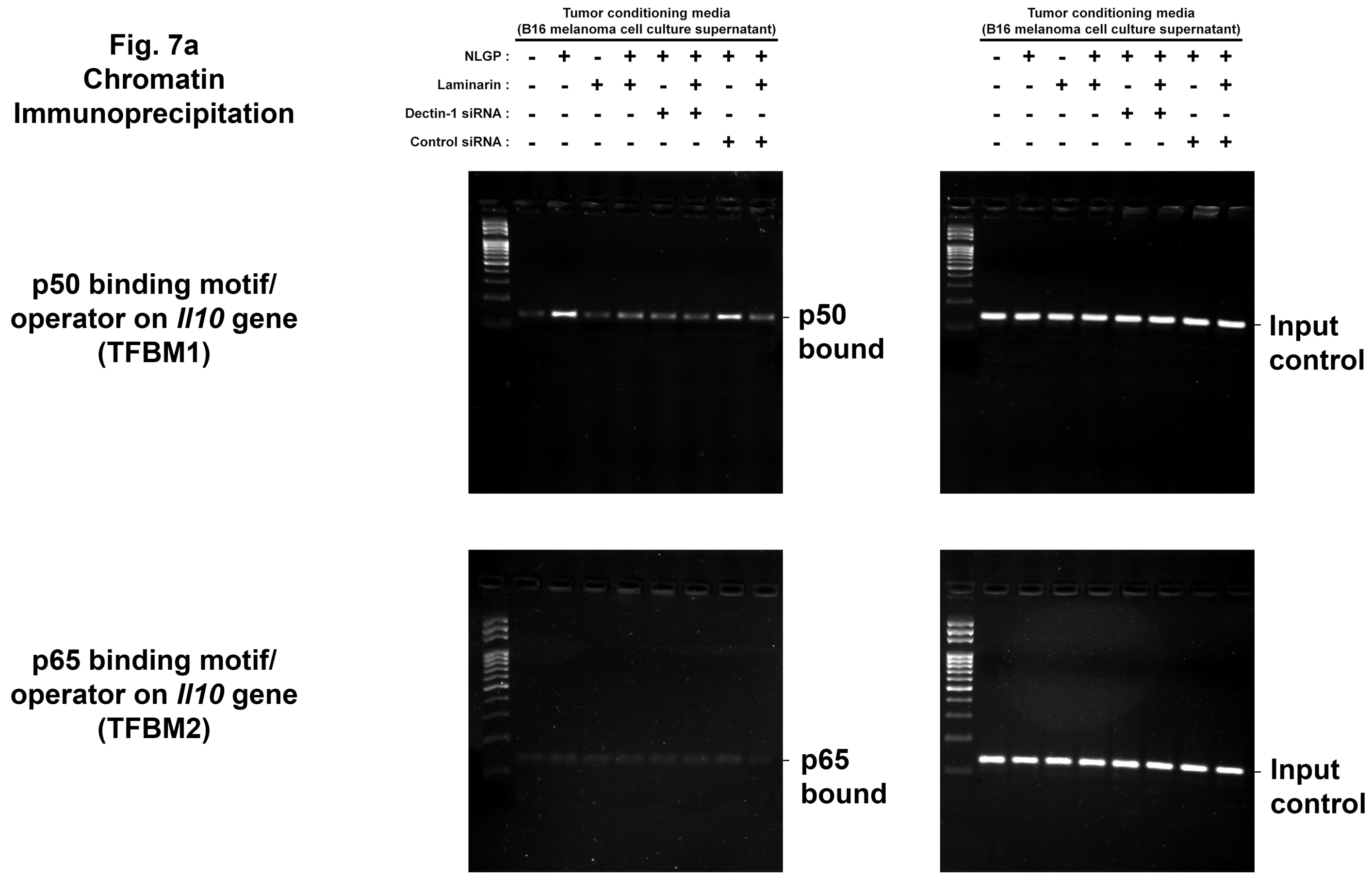

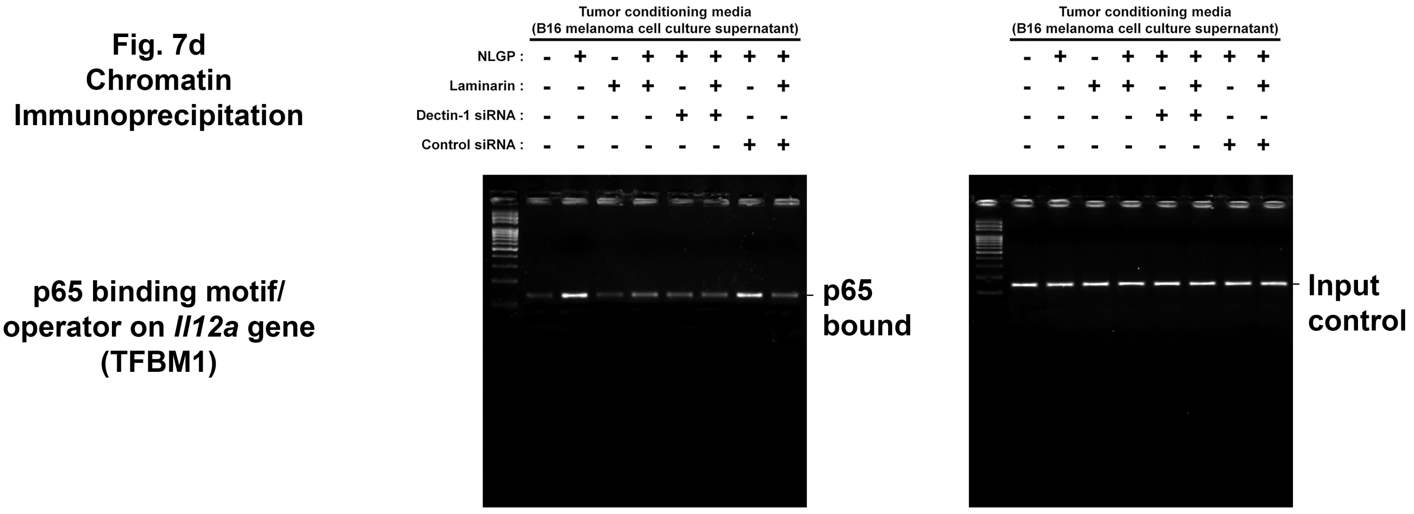

Fig. 7d
Chromatin Immunoprecipitation

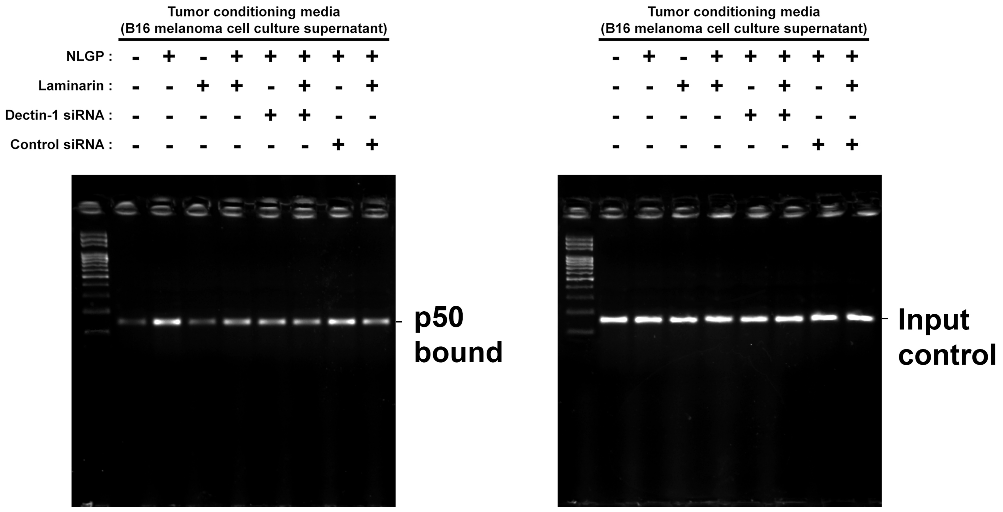

p50 binding motif/ operator on *Il12a* gene (TFBM2)
